# Water-resistant perovskite nanodots enable robust two-photon lasing in aqueous environment

**DOI:** 10.1038/s41467-020-15016-2

**Published:** 2020-03-04

**Authors:** Siqi Li, Dangyuan Lei, Wei Ren, Xuyun Guo, Shengfan Wu, Ye Zhu, Andrey L. Rogach, Manish Chhowalla, Alex K.-Y. Jen

**Affiliations:** 1 Department of Applied Physics, The Hong Kong Polytechnic University, Hung Hom, SAR Hong Kong; 2 Department of Materials Science and Engineering, and Centre for Functional Photonics, City University of Hong Kong, 83 Tat Chee Avenue, Kowloon, SAR Hong Kong; 3 Department of Materials Science and Engineering, and Department of Chemistry, City University of Hong Kong, 83 Tat Chee Avenue, Kowloon, SAR Hong Kong; 4 Department of Materials Science and Metallurgy, University of Cambridge, Cambridge, CB2 1TN UK

**Keywords:** Materials for optics, Lasers, LEDs and light sources

## Abstract

Owing to their large absorption cross-sections and high photoluminescence quantum yields, lead halide perovskite quantum dots (PQDs) are regarded as a promising candidate for various optoelectronics applications. However, easy degradation of PQDs in water and in a humid environment is a critical hindrance for applications. Here we develop a Pb-S bonding approach to synthesize water-resistant perovskite@silica nanodots keeping their emission in water for over six weeks. A two-photon whispering-gallery mode laser device made of these ultra-stable nanodots retain 80% of its initial emission quantum yield when immersed in water for 13 h, and a two-photon random laser based on the perovskite@silica nanodots powder could still operate after the nanodots were dispersed in water for up to 15 days. Our synthetic approach opens up an entirely new avenue for utilizing PQDs in aqueous environment, which will significantly broaden their applications not only in optoelectronics but also in bioimaging and biosensing.

## Abstract (web summary)

Lead halide perovskite quantum dots (PQDs) promise applications in optoelectronics but are limited by sensitivity to wet environments. Here the authors develop a Pb-S bonding approach to synthesize PQDs@silica nanodots that are capable of emitting and lasing in aqueous environments for long periods.

## Introduction

Lead halide perovskite quantum dots (PQDs)^1^ have emerged to be a promising active material system for optoelectronics devices such as light-emitting diodes (LEDs)^2^, solar cells^3^, scintillators^4^, and lasers^5^. Notably, both amplified spontaneous emission (ASE) and lasing have recently been demonstrated for PQDs at low thresholds under either one-photon or multi-photon pumping^5–10^. Compared to traditional semiconductor QDs-based lasers (e.g., CdSe/ZnS QDs), the PQDs-based lasers show much higher quality factors under two-photon pumping and a significantly reduced pumping threshold^6^. In particular, two-photon lasing can be potentially applied for in-vivo photodynamic therapies, owing to the large penetration depth of the near-infrared/infrared excitation^11–13^. However, it is still a drawback of existing PQDs that they would lose their structural integrity and emission ability when exposed to aqueous medium or even in humid air^14,15^. Therefore, combating the water-instability of PQDs has been a top priority. However, even numerous attempts have been tried to synthesize PQDs directly in water, they only resulted in limited stability so far^16^.

Enormous efforts have thus been devoted to enhancing stability of PQDs by functionalizing them with moisture-tolerance molecules^17^, depositing passivation layers onto the PQDs films^18^, coating metal shells^19^, and encapsulating them into polymer microspheres^20,21^. These strategies have improved the stability of PQDs in humid environment to some extent; nevertheless, all these methods still have their own drawbacks that hinder the practical applications of PQDs. For example, the modifications with moisture-tolerance surfactant molecules and passivation layers cannot provide long persistence of the PQDs in aqueous medium; the coating of metal shells requires high temperatures, which would inevitably degrade the inherent optical properties of the PQDs; and the encapsulation of PQDs within microspheres will significantly increase their total sizes, incurring difficulties in coupling them with conventional optoelectronics platforms such as optical cavities for laser devices. Thus, it remains highly desirable to endow the PQDs with water-resistant capabilities without significantly increasing their sizes or compromising their attractive optical features in developing PQDs-based two-photon lasers.

Herein, taking advantage of strong Pb–S bonding, we developed a facile approach to produce water-resistant PQDs@SiO_2_ nanodots (wr-PNDs). Superior to the previously reported powdered PQDs-silica nanocomposites that only exhibited reasonable stability in humid air^22,23^, our wr-PNDs could be easily dispersed in water and keep emission for up to six weeks, evidencing on an unprecedented water-resistance capability. This allowed us to realize a two-photon whispering-gallery mode laser device by introducing the nanodots into a capillary-like whispery gallery microcavity and a two-photon random laser device assembled from the wr-PNDs powder. Compared with conventional PQDs-based lasers, the two devices showed a great improvement in water-stability while keeping a low threshold and a high quality factor, potentially allowing their application in a biological medium.

## Results

### Encapsulating PQDs in a thiol-functionalized silica shell

**Fig. 1:**
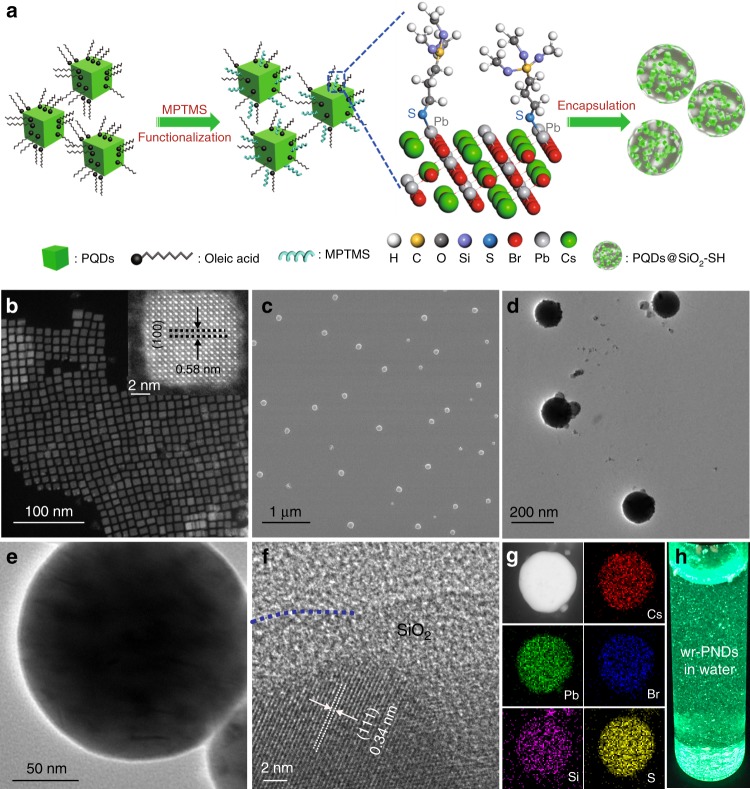
Synthesis and characterization of wr-PNDs. **a** Schematics of the synthetic procedure for making wr-PNDs. **b** HAADF-STEM micrographs of CsPbBr_3_ PQDs. **c** SEM and **d**, **e** TEM micrographs of wr-PNDs. **f** HRTEM of a PQD encapsulated within a silica shell. **g** HAADF-STEM and EDX elemental mapping images of a typical wr-PND. **h** Photograph of fresh wr-PNDs dispersed in water under UV light illumination.

CsPbBr_3_ PQDs were synthesized through a well-documented protocol^24^, and used as building blocks for the subsequent synthesis of wr-PNDs following the procedures depicted in Fig. 1a. High-angle annular dark-field scanning transmission electron microscope (HAADF-STEM) micrographs (Fig. 1b) show that pristine PQDs are in cubic phase, with a lattice constant of ~0.58 nm and an average edge length of ~9.5 nm (see the histogram statistics in Supplementary Fig. 1a). To produce wr-PNDs, the pristine PQDs were dispersed in a mixed solvent of toluene and (3-mercaptopropyl)trimethoxysilane (C_6_H_15_O_3_Si-SH, MPTMS), and de-ionized water were added to initiate the hydrolysis of MPTMS. MPTMS molecules could be tightly adsorbed onto the PQDs due to the formation of Pb-S bonding^25^. These bounded molecules decrease the interfacial energy between the PQDs and silica by providing additional steric stabilization, thereby avoiding the phase separation between the PQDs and silica. With proceeding of hydrolysis and condensation of MPTMS triggered by the added water, silica-encapsulated PQDs-Pb–S–SiO_2_-SH nanodots were produced. The detailed information about their fabrication can be found in the Method section.

Different from the previous report in which tetraethyl orthosilicate (TEOS) or tetramethyl orthosilicate (TMOS) was selected as silica source^22^, the silane molecules first physically adsorb on the surface of PQDs, then hydrolyze and condense to form silica. As a result, it is energetically favorable to produce phase-separated heteroparticles because this minimizes the interfacial area between the two components^26^. The TEM micrographs in Supplementary Fig. 2 further show that during the silica-encapsulation process, thin silica layers with thicknesses of several nanometers were first coated onto the surface of the PQDs (in about 1 h). After longer reaction time (about 6 h), some larger SiO_2_ nanoparticles were observed, and more PQDs were embedded in these nanoparticles. When the encapsulation time reached 32 h, the final products have an average size of about 170 nm (Fig. 1c, d and Supplementary Fig. 1b), which is small enough for device integration. The TEM image (Supplementary Fig. 3) confirms that multiple PQDs were incorporated in the silica matrix while maintaining their cubic morphology, and the high-resolution transmission electron microscopy (HRTEM) indicates that the PQDs remained in the cubic crystalline structure (Fig. 1f). HAADF-STEM and energy-dispersive X-ray (EDX) element mapping images of a typical wr-PND are shown in Fig. 1g, with the Cs, Pb, Br, S, and Si elements found to be homogeneously distributed over the entire volume of the nanodot, confirming that the PQDs were successfully and uniformly encapsulated into the silica matrix. Figure 1h shows that dried wr-PNDs powder could easily form an aqueous suspension, which emitted strong green light even after six weeks of storage (Supplementary Fig. 4).

### Analysis of Pb-S bonding between the PQDs and the silica shell

**Fig. 2:**
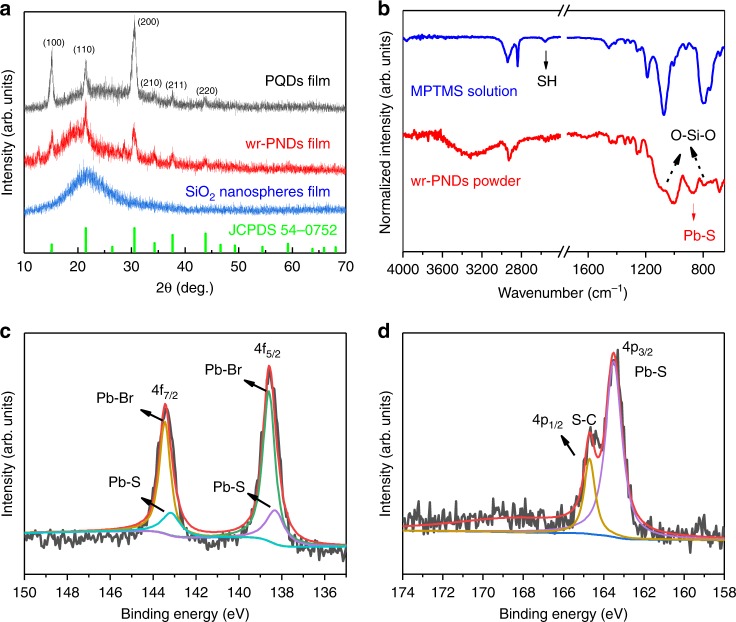
Spectroscopic verification of the Pb-S bonding between the PQDs and silica shell. **a** Powder XRD spectra of wr-PNDs, PQDs, and SiO_2_ nanospheres. **b** FTIR spectra of wr-PNDs powder (red), MPTMS solution (blue). **c**, **d** XPS of Pb (4f) and S (4p) regions in wr-PNDs. Source data are provided as a Source Data file.

The crystallinity of wr-PNDs was studied by X-ray diffraction (XRD) measurement. The XRD patterns in Fig. 2a reveal that the PQDs belong to cubic phase (*a* = 5.605 Å, space group Pm3̅m, JCPDS75-0412) before and after silica coating. The broad peak in the range between 15^o^ and 35^o^ indicates the existence of amorphous silica in the wr-PNDs. Fourier-transform infrared spectroscopy (FTIR) spectra in Fig. 2b testify to the appearance of symmetric stretching vibration and asymmetric vibration of (Si–O–Si) at about 800 and 1085 cm^−1^ in the spectrum of the wr-PNDs^27^, while they are absent in the spectrum of the pristine PQDs (Supplementary Fig. 5a). The peak at about 880 cm^−1^ is attributed to the vibration mode of Pb–S bonding^28^. X-ray photoelectron spectroscopy (XPS) analyses were performed as well; Supplementary Fig. 6a shows the full-scan XPS spectrum of the wr-PNDs, where signals related to Cs, Pb, Br, S, Si, and O are observed above the detection limits. In the high-resolution spectrum of the Pb 4 f region (Fig. 2c), the Pb 4f_7/2_ and 4f_5/2_ are deconvoluted into two components: the peaks at 138.6 eV and 143.5 eV correspond to the Pb-Br bond^29^, while the peaks at 138.3 eV and 143.2 eV correspond to the Pb–S bond^30^. Figure 2d indicates that the Pb-S bonding also exists in the high-resolution XPS of the S 4p region. The peak at 163.5 eV belongs to the Pb-S bond^30^ and the peak at 164.7 eV originates from the C–S bond of MPTMS^31^.

### Evaluation of the water resistance and optical properties of wr-PNDs

**Fig. 3:**
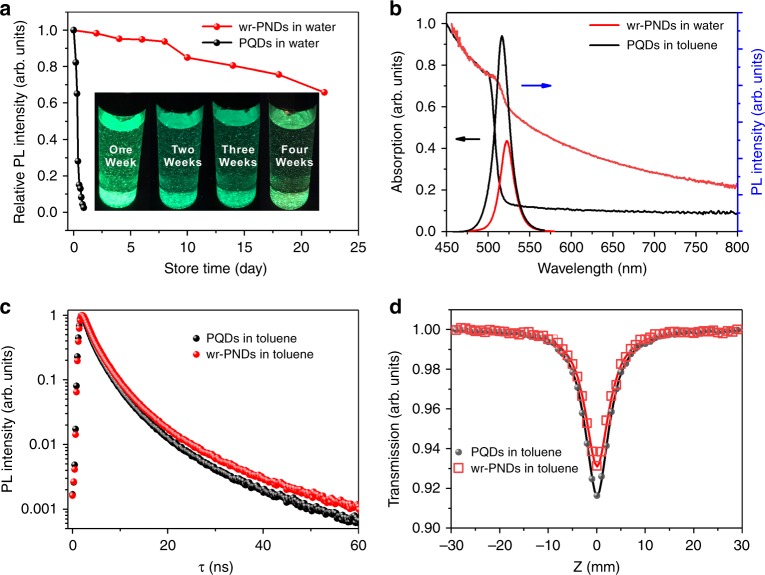
Comparison of optical properties between wr-PNDs and PQDs. **a** Relative PL intensities of wr-PNDs and PQDs stored in water for different periods of time. **b** UV-visible absorption and PL spectra of PQDs in toluene and wr-PNDs in water. **c** Time-resolved PL spectra of PQDs and wr-PNDs in toluene. **d** Open-aperture z-scan curves measured on the toluene solution of PQDs and wr-PNDs. Source data are provided as a Source Data file.

To quantify the emission characteristics of the PQDs, their photoluminescence quantum yields (PLQYs) were tracked during the synthesis process by using a fluorescence spectrometer equipped with an integrating sphere (Supplementary Fig. 7). The PLQY of the wr-PNDs was close to that of the pristine PQDs (78% vs. 89%) after silica coating, demonstrating their good structural integrity. To quantify their water-resistant capabilities, the two samples were dispersed in water and their relative PL intensities were tracked with the same fluorescence spectrometer system. As seen from Fig. 3a, the relative PL intensity of the pristine PQDs powder in water dropped to nearly zero rapidly, whereas the wr-PNDs retained 50% of the initial PL intensity after storing in water for over 20 days. The absorption spectra in Fig. 3b show that the bandgap energy of the wr-PNDs in water is slightly red-shifted compared to the pristine PQDs in toluene (2.38 eV vs. 2.4 eV, Supplementary Fig. 5b). Under the same excitation power, both emission spectra have a full width at half maximum (FWHM) of ~21 nm. Similar absorption and emission characteristics of the wr-PNDs originate from the pristine PQDs.

The time-resolved PL spectra of the PQDs and wr-PNDs in toluene were also measured (Fig. 3c), and they can be well fitted by a double-exponential decay function^32,33^, *I* = *I*_1_exp(−*t*/*τ*_1_) + *I*_2_exp(−*t*/*τ*_2_). This indicates two components in the recombination process: a dominating fast component *τ*_1_, which is attributed to the intrinsic photon-radiative recombination, and a slower component *τ*_2_, which is attributed to the shallow-level surface trap assisted recombination^33^. The average lifetime *τ*_aver_ can thus be given by $\tau _{{\mathrm{aver}}} = (I_1\tau _1^2 + I_2\tau _2^2)/(I_1\tau _1 + I_2\tau _2)$, where *I*_1_ and *I*_2_ represent the respective proportion of radiative and non-radiative transitions in the recombination process. The fitting results in Supplementary Table 1 show that the non-radiative lifetime *τ*_2_ is longer in the wr-PNDs (12.4 ns) than in the pristine PQDs (8.1 ns), and its proportion decreases from 12 to 6%, testifying that the shallow-level non-radiative recombination is suppressed by the surface passivation using silica shell^34^. Open-aperture *Z*-scan measurement results in Fig. 3d (experimental setup sketched in Fig. 8) show that the two-photon absorption coefficient only slightly decreases from ~0.092 cm/GW for the pristine PQDs to ~0.074 cm/GW for the wr-PNDs, which is comparable to the absorption coefficient of CsPbBr_3_ PQDs in previous report^35^. Both prolonged PL lifetimes and the large two-photon absorption coefficient of the perovskite nanodots are beneficial for achieving resonant two-photon lasing.

### Realization of wr-PNDs based lasers

To better comprehend the performances of wr-PNDs based lasers, ASE spectra of wr-PNDs and PQDs films were measured at first. Thin films of pristine PQDs and wr-PNDs on quartz substrate were prepared by spin-coating. As measured from their cross-sectional SEM images (Supplementary Fig. 9), the thicknesses of both films are about 450 nm. To demonstrate ASE, the two samples were pumped at 800 nm in a standard stripe pumping configuration equipped with a femtosecond laser (Libra Coherent, 1 kHz, 50 fs, inset of Supplementary Fig. 10a). Supplementary Fig. 10a shows PL spectra of the wr-PNDs film under varied pumping intensity. PL spectra under low pump intensities (<~1.23 mJ cm^−2^) are dominated by a relatively broad spontaneous emission peak located at 527 nm (FWHM ≈18.5 nm). When the pump intensity exceeds ~1.29 mJ cm^−2^, a much narrower peak (FWHM ≈5 nm, Supplementary Fig. 10b) emerges, indicating the occurrence of frequency-upconverted stimulated emission under a two-photon absorption process. The ASE peak is red-shifted by ~8 nm with respect to the center of broad PL spectra, which arises from the bi-excitonic recombination in PQDs^8^. As a comparison, the emission and threshold behavior of pristine PQDs were also examined as shown in Supplementary Fig. 10c and d. The threshold of the pristine PQDs film is almost the same as that for the wr-PNDs film (1.16 vs. 1.29 mJ cm^−2^), demonstrating the inheritance of excellent gain response from the PQDs.

**Fig. 4:**
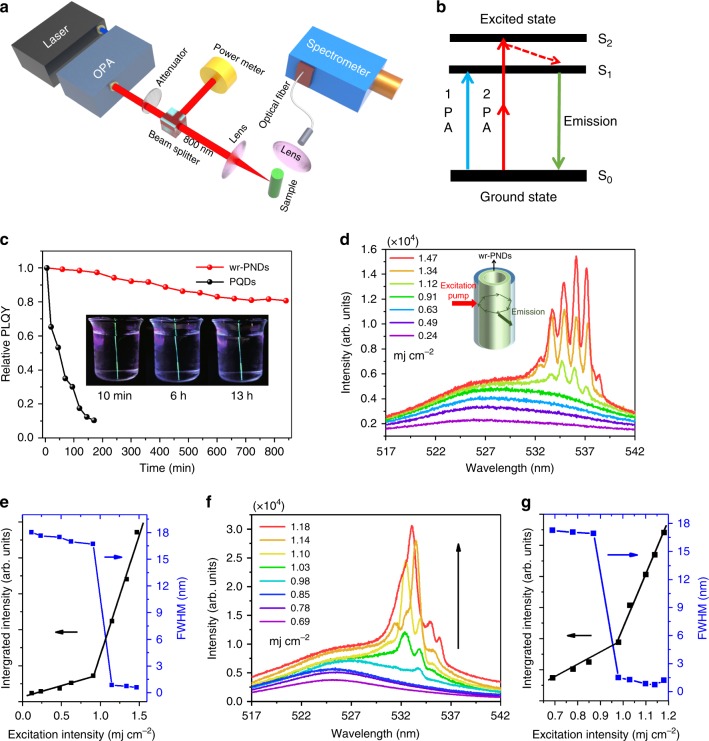
wr-PNDs based water-proof two-photon pumped lasers. **a** Pumping configuration used in two-photon lasing measurements. **b** Jablonski diagram of the two-photon absorption and emission in wr-PNDs and PQDs. **c** Relative PLQYs of the laser devices (measured in an integrating sphere under 375 nm laser excitation) based on wr-PNDs (red dots) and pristine PQDs (black dots), which were immersed in water for different periods of time. The insets are the photographs of the wr-PNDs-based laser in water taken at different time under UV light illumination. **d** Emission spectra and **e** integrated intensity and emission FWHM for the wr-PNDs-based laser immersed in water for 13 h, as a function of pumping intensity under 800 nm fs laser excitation. The insert in **d** sketches the capillary-like microcavity laser device. **f** Emission spectra and **g** integrated intensity of the wr-PNDs-based random laser device stored in water for 15 days. Source data are provided as a Source Data file.

A two-photon pumped laser was fabricated by introducing the wr-PNDs in a capillary-like whispery gallery microcavity (see Methods for details), and its narrowed ASE and lasing response under 800 nm fs laser excitation (Libra Coherent, 1 kHz, 50 fs) was investigated with the setup shown in Fig. 4a (detailed description of optical measurements is given in Supplementary Information). Since the energy of 800 nm laser photons is far below the bandgap energy of the wr-PNDs, here simultaneous two-photon absorption occurs and gives rise to frequency-upconverted emission, as schematically illustrated in Fig. 4b. Firstly, the water-resistance performance of the wr-PNDs-based laser device was compared with a similar device made of the pristine PQDs. As shown in Fig. 4c, the relative PLQY of the wr-PNDs-based device under 375 nm laser excitation retained 80% of its initial value after immersion in water for 13 h, whereas the PLQY of the pristine PQDs-based device decreased to less than 10% after 3 h in water. The robust water stability of the wr-PNDs-based device was further elaborated by the photographs of the device in the inset of Fig. 4c. Then, pumping-dependent emission spectral characteristics of the immersed wr-PNDs-based laser was investigated in order to unravel its frequency-upconverted lasing mechanism through comparison with the thick wr-PNDs planar film. As shown in Fig. 4d, the emission from the laser device exhibits a broad PL peak with intensity increasing slowly when the pumping intensities are below 0.91 mJ cm^–2^. Further increase of pumping density leads to the emergence of gain-induced sharp peaks (with a line width of 0.3–0.5 nm) decorated on a narrowed ASE peak of the laser device, whereas the film device only exhibits a narrowed ASE peak (Supplementary Fig. 10). The lasing character of the capillary-cavity device was further evidenced by the pronounced threshold behavior in the power dependence of integrated emission intensity (Fig. 4e). The lasing threshold for this device was estimated to be ~1.12 mJ cm^−2^, and the quality factors (Q) of the associated lasing modes are in the range of 1070–1700, which are several folds larger than the reported devices made of CH_3_NH_3_PbBr_3_ microdisks (~430)^36^ and microwires (~682)^37^. The high Q factors and evenly distributed lasing peaks shown in Supplementary Fig. 11 correspond to the whispering gallery modes (WGM) arising from the total internal reflection at the interface between the tubular microcavity and the wr-PND layer. By applying the WGM theory^6^, the lasing spikes are assigned to mode numbers indexed from 484 to 491. Finally, the polar of plot of output intensity as a function of detection polarization in Supplementary Fig. 12 shows that the lasing emission is linearly polarized.

To further verify the water-resistance capability of the wr-PNDs, the wr-PNDs powder which had been dispersed in water for 15 days were collected, and its lasing properties were checked. Different from the WGM laser made by incorporating the wr-PNDs in the microcavity as demonstrated above, the powder sample itself can work as a random laser without the need of optical coupling with an external cavity resonator^38^. In general, random lasing does not require a well-confined laser cavity but relies on multiple light scattering in a disordered scattering medium^39–41^. As a result, random lasers have lower coherence than the lasers based on optical cavities, thus ensuring their application in speckle-free imaging and bioimaging^42^. Randomly distributed wr-PNDs in the powder can provide effective scattering centers for random lasing. When the wr-PNDs powder is pumped by the 800 nm fs laser at 173 K, a broad spontaneous emission band centered at ~525 nm is observed under low-power excitation (Fig. 4f). With an increase of pump power, distinct interference features (spectral spikes) gradually emerges on the top of the emission peak. Unlike the ASE and WGM lasing demonstrated above, the wavelengths and intensities of the spikes vary from spectrum to spectrum, evidencing on the random lasing nature with coherent optical feedback.

## Discussion

In conclusion, we synthesized wr-PNDs by embedding pristine CsPbBr_3_ PQDs into water-stable SiO_2_-SH matrix. The wr-PNDs dispersed in water persistently emit green light for six weeks, whereas the pristine PQDs in water become quenched soon. Such remarkable water-stability enables the realization of a frequency-upconverted laser device: a two-photon WGM laser device retains 80% of its initial value after immersion in water for 13 h. The device exhibits lasing at low threshold and high Q factor under two-photon pumping through coupling the amplified spontaneous emission of PQDs to the whispering gallery modes of a tubular microcavity. Furthermore, a two-photon-pumped random laser device based on the perovskite@silica nanodots powder could still operate after the nanodots were dispersed in water for up to 15 days. The water-proof capability and outstanding optical properties make the wr-PNDs an ideal active material system for a variety of photonics and optoelectronics applications not only in humid air but even in an aqueous medium, which will significantly extend the application scenario of PQDs in solid-state laser devices, and also as bioimaging/biosensing fluorescent labels.

## Methods

### Reagents

Oleic acid (OA), octadecene (ODE), oleylamine (OAm), cesium carbonate (Cs_2_CO_3_), lead bromide (PbBr_2_), (3-mercaptopropyl) trimethoxy silaneare (MPTMS), methyl acetate, and toluene were purchased from Aladdin and used without further purification.

### Synthesis of CsPbBr_3_ PQDs

The synthesis follows the modified method reported by Wang et al.^43^. A mixture of 0.267 g Cs_2_CO_3_, 0.833 mL of OA, and 10 mL of ODE was degassed under argon flow in a 50 mL four-neck flask at 130 °C for 10 min. The temperature was raised to 150 °C for another 10 min, until all the Cs_2_CO_3_ reacted with OA. After naturally cooling down to room temperature, the Cs-precursor was kept in a glove box. ODE (10 mL), OA (1 mL), OAm (1 mL), and 0.138 g PbBr_2_ were mixed and degassed in N_2_ at 130 °C for 1 h. After complete dissolution of PbBr_2_ salt, the temperature was raised to 180 °C and kept for another 10 min. Then, 1 mL of the Cs-precursor was swiftly injected into the above hot mixture and the reaction was stopped with ice bath after 5 s. The crude solution, which was centrifuged and the precipitate was discarded. The CsPbBr_3_ PQDs from supernatant were precipitated with methyl acetate, by centrifugation at 13,000 rpm for 5 min. The obtained PQDs were then washed by toluene and methyl acetate again, finally re-dispersed in 4 mL toluene for further use.

### Synthesis of water-resistant perovskite nanodots (wr-PNDs)

Two mililiter of the precursor PQDs in toluene (~0.046 g mL^−1^) was mixed with 5 µL MPTMS, and the resultant solution was sonicated and stirred vigorously. After silanization for 1 h, the crude solution was transferred to a 15 mL toluene with another 20 µL MPTMS added, followed by adding 2 µL of deionized water slowly to initiate the hydrolysis of MPTMS. The solution was stirred at the temperature 45 °C to promote the hydrolysis and condensation of MPTMS. After stirring for 32 h, the precipitate was collected by centrifugation, dried and dispersed in water for water-stability examination, and in toluene for the control studies.

### Fabrication of ASE films and laser devices

ASE films were produced by spin-coating PQDs or wr-PNDs both in toluene (with 5% PMMA) on plasma-cleaned glass substrates. Laser devices were fabricated by immersing a glass tube (inner radius ~40 μm) into a highly-concentrated PQD solution or into a wr-PND solution. The tubes filled with these solutions were left in vacuum overnight to evaporate the solvent, which resulted in a thin layer of wr-PNDs coating on the inner wall of each tube.

### Characterization

Transmission electron microscopy (TEM) was carried out on a JEM-2100 electron microscope, operating at an acceleration voltage of 100 kV. Scanning transmission electron microscopy (STEM) was carried out on a JEM-2100F field-emission electron microscope, operating at an acceleration voltage of 200 kV. SEM images are captured with a JEOL Field Emission SEM. The concentration of the precursor PQDs solution was calibrated with reference to the mass of lead measured by an inductive coupled plasma optical emission spectrometer (Agilent 710). X-ray diffraction (XRD) patterns were acquired with a Bruker AXS D2 phaser X-ray diffractometer equipped with a Cu Kα radiation source (*λ* = 1.54 Å) at 40 kV and 30 mA. Infrared absorption spectroscopy was carried out on a BRUKER Vertex Fourier Transform Infrared spectrometer. UPS and XPS measurements were carried out on a VG ESCALAB 220i-XL surface analysis system equipped with a He discharge lamp (hν = 21.2 eV) and a monochromatic Al–Kα X-ray gun (hν = 1486.6 eV).

### Optical measurements

Absorption spectra were obtained on a Shimadzu UV2550 spectrophotometer. Steady-state and time-resolved PL spectra were collected on an FLS920 fluorescence spectrometer (Edinburgh Instruments) under the excitation by a 375 nm pulsed diode laser (pulse width 75 ps). PLQY measurements were performed in an integrating sphere (Edinburgh Instruments) under the excitation of a 375 nm Xe lamp. For Z-scan measurements (Supplementary Fig. 8), the excitation pulse (50 fs, 1 kHz) was deduced from a Ti:sapphire femtosecond laser (Coherent Libra) integrated with an optical parametric amplifier (Coherent OPerA Solo). The laser light was split into two beams through a beam splitter. A thin quartz cuvette (1 mm thickness) was chosen to satisfy the widely-used approximation proposed by Sheikbahae et al.^44^. The transmitted beam was focused onto the surface of the sample to a minimum size spot of ≈40 μm by a convex lens (lens 1 in Supplementary Fig. 8) with a focal length of 10 cm. The beam transmitted through the sample was collimated to the detector 1. The sample was allowed to move between lens 1 and lens 2. Any fluctuation of the laser light could be detected by detector 2. For ASE and lasing measurements, the excitation source was the same as the Z-scan laser. The excitation beam integrated with an optical parametric amplifier was focused onto the surface of the samples by a lens. Light emitted from the surface of the samples was collected by an optical fiber coupled to a Princeton Spectra Pro 2750 monochromator integrated with a ProEM EMCCD camera with a spectral resolution 0.0163 nm.

## Supplementary information

Supplementary Information

## Data Availability

The data that support the plots within this paper and other findings of this study are available from the corresponding authors upon reasonable request.

## Source Data

Source Data
