# Supplementary material for: Water-resistant perovskite nanodots enable robust two-photon lasing in aqueous environment
Source: Nat Commun. 2020 Mar 4;11:1192. doi: 10.1038/s41467-020-15016-2 (PMC7055291; doi:10.1038/s41467-020-15016-2)
Supplement: Supplementary file 1 — Supplementary Information [file 41467_2020_15016_MOESM1_ESM.docx]

**Supplementary Information**

**Water-Resistant Perovskite Nanodots Enable Robust Two-Photon** **Lasing in Aqueous Environment**

Siqi Li^1^, Dangyuan Lei^2,1,*^, Wei Ren^1^, Xuyun Guo^1^, Shengfan Wu^3^, Ye Zhu^1^, Andrey L. Rogach^2^, Manish Chhowalla^4^, Alex K.-Y. Jen^3^

^1^Department of Applied Physics, The Hong Kong Polytechnic University, Hong Kong S.A.R.

^2^Department of Materials Science and Engineering, and Centre for Functional Photonics, City University of Hong Kong, 83 Tat Chee Avenue, Kowloon, Hong Kong S.A.R.

^3^Department of Materials Science and Engineering, and Department of Chemistry, City University of Hong Kong, 83 Tat Chee Avenue, Kowloon, Hong Kong S.A.R.

^4^Department of Materials Science & Metallurgy, University of Cambridge, Cambridge, UK

*Correspondence to: [dangylei@cityu.edu.hk](mailto:dangylei@cityu.edu.hk)

**

**

**Supplementary Figure 1** Size distribution histograms of **(a)** CsPbBr_3_ PQDs and **(b)** wr-PNDs.

**
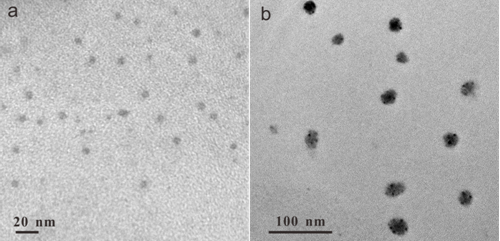
**

**Supplementary Figure 2** TEM micrographs of wr-PNDs during the process of silica encapsulation for 1h **(a)** and 6 h **(b),** respectively.


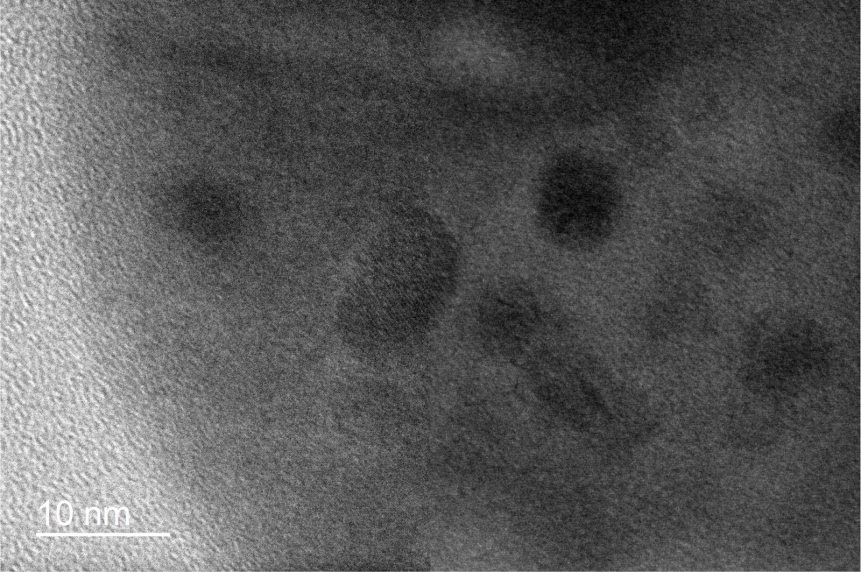


**Supplementary Figure 3** HRTEM image of PQDs embedded in the silica matrix.


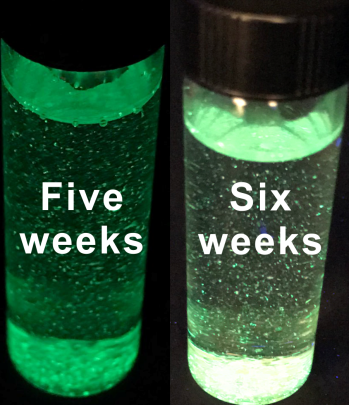


**Supplementary Figure 4** Photographs of wr-PNDs powders dispersed in aqueous suspension for five (left) and six (right) weeks.





**Supplementary Figure 5** **(a)** FTIR spectrum of the PQDs powder. The footprint vibrational modes at 1645 and 1552 cm^-1^ can be attributed respectively to C=C and C=O stretching peaks, which are the characteristic infrared absorption peaks of oleic acid adsorbed on the surface of PQDs. **(b)** The bandgap of wr-PNDs and PQDs.





**Supplementary Figure 6 (a)** Full-scan XPS spectrum of wr-PNDs powder. High-resolution XPS surveys of **(b)** Pb 4f region for pristine PQDs, **(c, d)** Cs 3d and **(e, f)** Br 3d regions for wr-PNDs and pristine PQDs, respectively.

**Supplementary Note 1** Supplementary Figure 6b shows the Pb 4f XPS spectrum for the pristine PQDs, where two peaks with binding energies of 138.6 eV (Pb 4f_7/2_) and 143.5 eV (4f_5/2_) can be observed in the fitting, both belonging to the Pb-Br bond[^1^](#_ENREF_1). Supplementary Figs. 6c and 6d show the Cs 3d spectra of the wr-PNDs and pristine PQDs, respectively. The spectra were fitted with two Gaussian peaks with binding energies at around 724 eV (3d_5/2_) and 738 eV (3d_3/2_)[^2^](#_ENREF_2), indicating no clear Cs-S bond formed. Supplementary Figs. 6e and 6f show that two XPS peaks corresponding to Br 3d_5/2_ and 3d_3/2_ appeared for the two samples at energies of 68.1 eV and 69.2 eV[^3^](#_ENREF_3), respectively.





**Supplementary Figure 7** Relative PLQY variation of PQDs during the silica coating process.


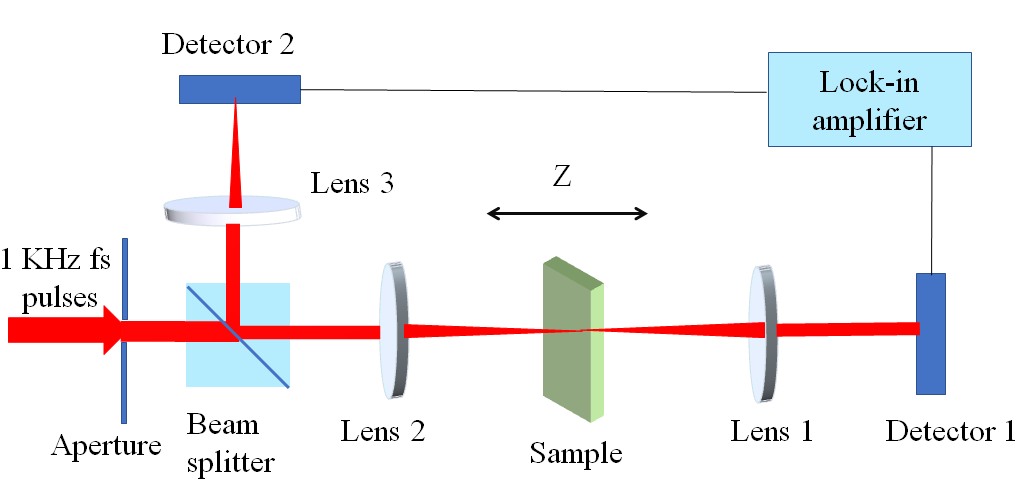


**Supplementary Figure 8** Experimental setup used for open-aperture Z-scan measurements.

**Supplementary Note 2 I**n open-aperture Z-scan measurements, the transmittance $T(z)$ of a sample in a two-photon absorption process can be expressed as

$$T\left( z \right)=\frac{1}{\sqrt{\pi}}\frac{1}{q(z)}\int_{-\infty}^{\infty} ln[1+q\left( z \right)\exp\left( -x^{2} \right)dx$$

where$q\left( z \right)=q_{0}/(1+{z^{2}}/{z_{0}^{2})}$*,*$q_{0}=\sigma^{(2)}NI_{0}L_{eff}/h\omega z_{0}={\pi\omega_{0}^{2}}/\lambda$ and$L_{eff}=\left\{ 1-\exp\left[ -\alpha_{0}L \right] \right\}/\alpha_{0}$. Here, $\sigma^{(2)}$, *N* and $\alpha_{0}$ are two-photon absorption cross section, the density, and the linear absorption of a sample$;\omega_{0}$ is the beam waist at the focal point, and $I_{0}$ and $\lambda$ are the peak intensity and wavelength of the incident beam, respectively.

**
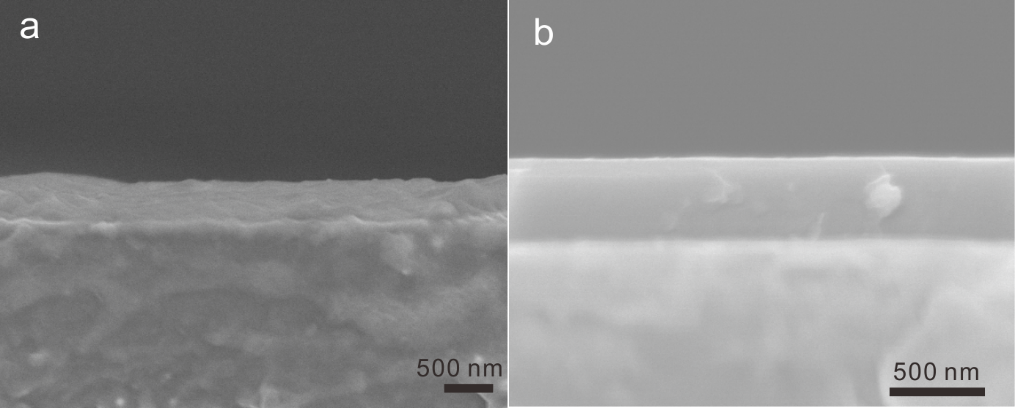
**

**Supplementary Figure 9** Cross-sectional SEM image of **(a)** a wr-PND film and **(b)** a pristine-PQD film, both deposited on plasma-cleaned glass substrates.





**Supplementary Figure 10** Emission spectra at varied pump fluence, integrated PL intensity and extracted FWHM for **(a, b)** a wr-PND film and **(c, d)** a pristine-PQD film.

**

**

**Supplementary Figure 11** Lasing modes calculated by the whispering-gallery-mode (WGM) theory. The resonant wavelength $\lambda_{m}$ is obtained by $m=\pi Dn/\lambda_{m}$, where $m$ is the mode number, *D* is the inner diameter of the capillary tube (~40 μm), and *n* is the refractive index of the perovskite material (~2.08)^4^.





**Supplementary Figure 12 Output polarization of WGM lasing.** The output polarization of our WGM microlaser was analyzed by placing a visible polarizer in front of the detector. The polarizer axis is parallel to the optical fiber (i.e. the rotation angle is zero). Integrated intensities of lasing peaks above the threshold as a function of rotation angle are plotted in the above polar plot, indicating that the lasing emission is nearly linearly polarized.





**Supplementary Figure 13** Normalized peak intensity of two-photon pumped lasing as a function of laser shots for a wr-PNDs-based laser under excitation by a 1 kHz femtosecond laser. The lasing intensity retained ~90% of its initial value after 10^6^ laser pulses (i.e. under fs excitation for about 1 h).

**Supplementary Table** 1. Fitting data of PL lifetimes for PQDs and wr-PNDs in toluene

|  | I_1_ | τ_1_ | I_2_ | τ_2_ | τ_aver_ |
| --- | --- | --- | --- | --- | --- |
| PQDs | 0.88 | 2.2 ns | 0.12 | 8.1 ns | 4.2 ns |
| wr-PNDs | 0.94 | 2.8 ns | 0.07 | 12.4 ns | 5.1 ns |

Supplementary References:

1 Pederson, L. *et al.* Two-dimensional chemical-state plot for lead using XPS. *J. Electron. Spectrosc. Relat. Phenom.* **28**, 203-209 (1982).

2 Zhang, M. *et al.* Stable CsPbBr_3_ perovskite quantum dots with high fluorescence quantum yields. *New. J. Chem.* **42**, 9496-9500 (2018).

3 Shi, Z.-F. *et al.* High-performance planar green light-emitting diodes based on a PEDOT: PSS/CH_3_NH_3_PbBr_3_/ZnO sandwich structure. *Nanoscale* **8**, 10035-10042 (2016).

4. Liu, Z.; Hu, Z.; Shi, T.;Du, J.; Yang, J.; Zhang, Z.; Tang, X.; Leng, Y., Stable and enhanced frequency up-converted lasing from CsPbBr3 quantum dots embedded in silica sphere. Opt. Express 2019, 27 (7), 9459-9466.
