# Supplementary figures and images for: Water-resistant perovskite nanodots enable robust two-photon lasing in aqueous environment
Source: Nat Commun. 2020 Mar 4;11:1192. doi: 10.1038/s41467-020-15016-2 (PMC7055291; doi:10.1038/s41467-020-15016-2)

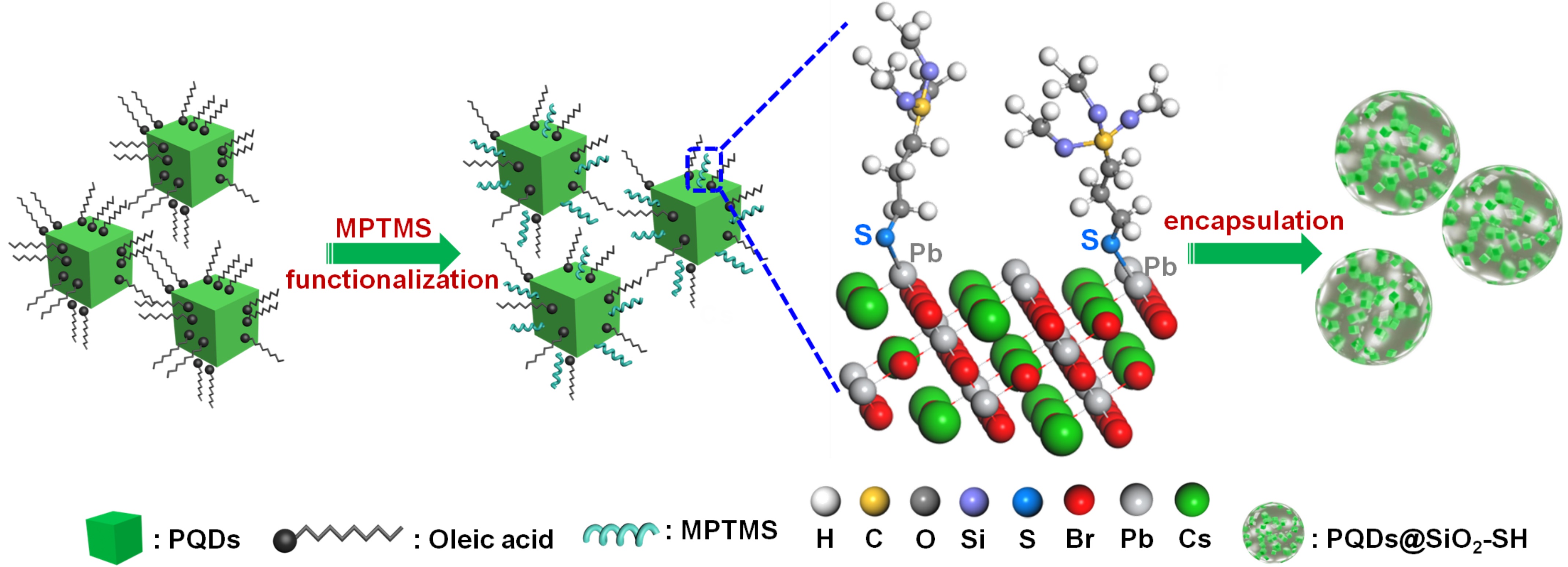

Supplement: Supplementary file 2 — Source Data [file 41467_2020_15016_MOESM2_ESM.zip › Source Data/Figure 1a.jpg]

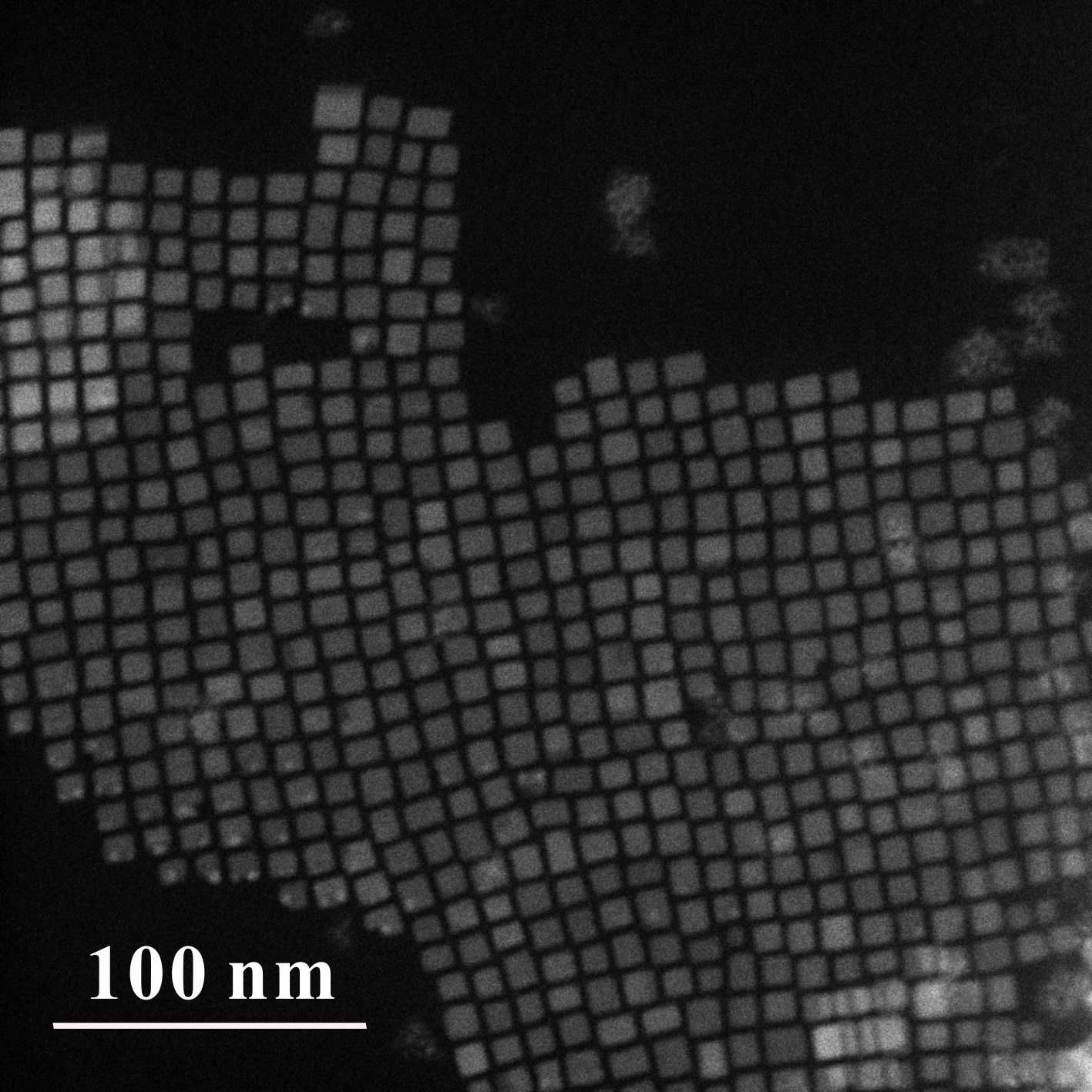

Supplement: Supplementary file 2 — Source Data [file 41467_2020_15016_MOESM2_ESM.zip › Source Data/Figure 1b.jpg]

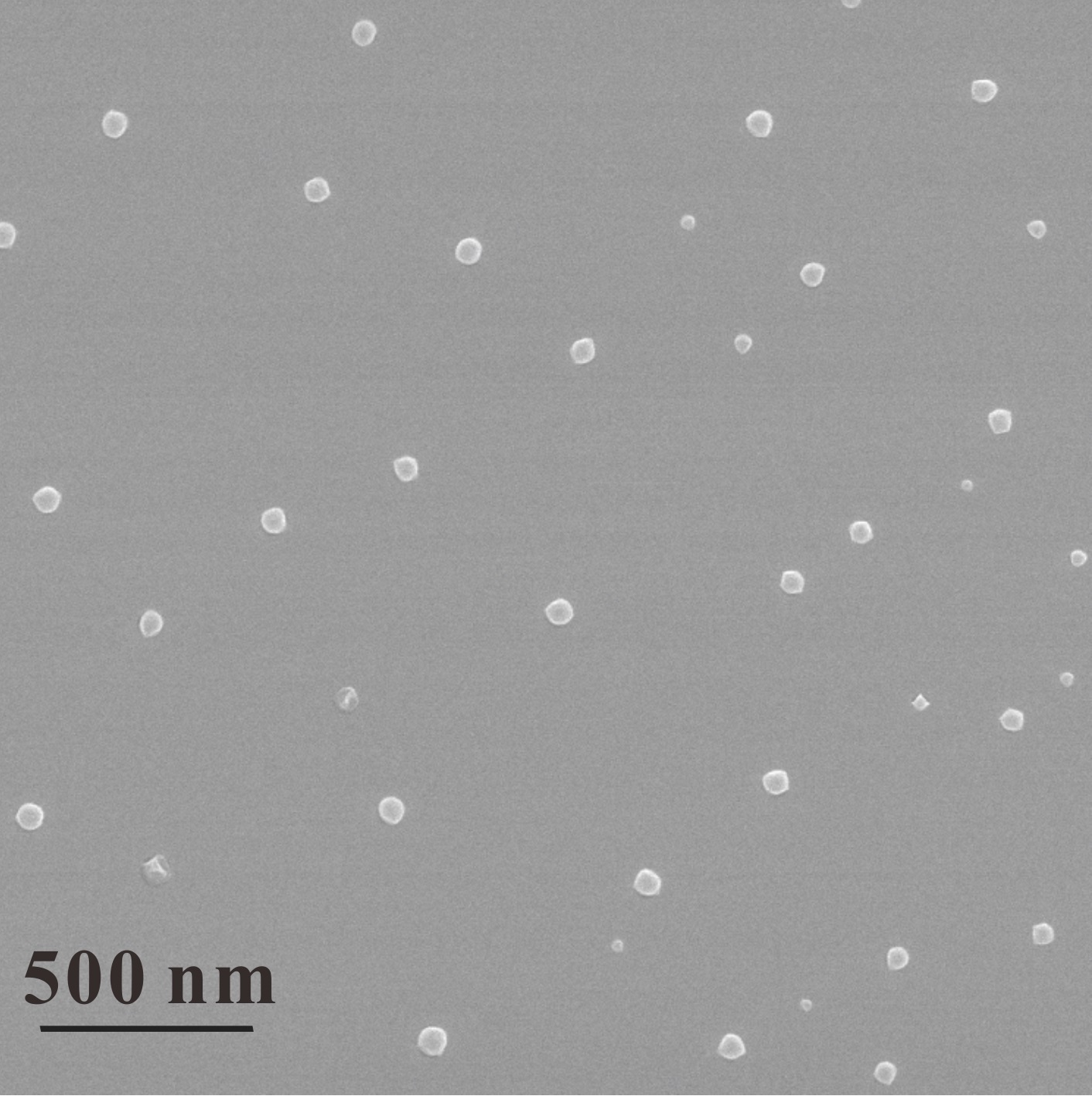

Supplement: Supplementary file 2 — Source Data [file 41467_2020_15016_MOESM2_ESM.zip › Source Data/Figure 1c.jpg]

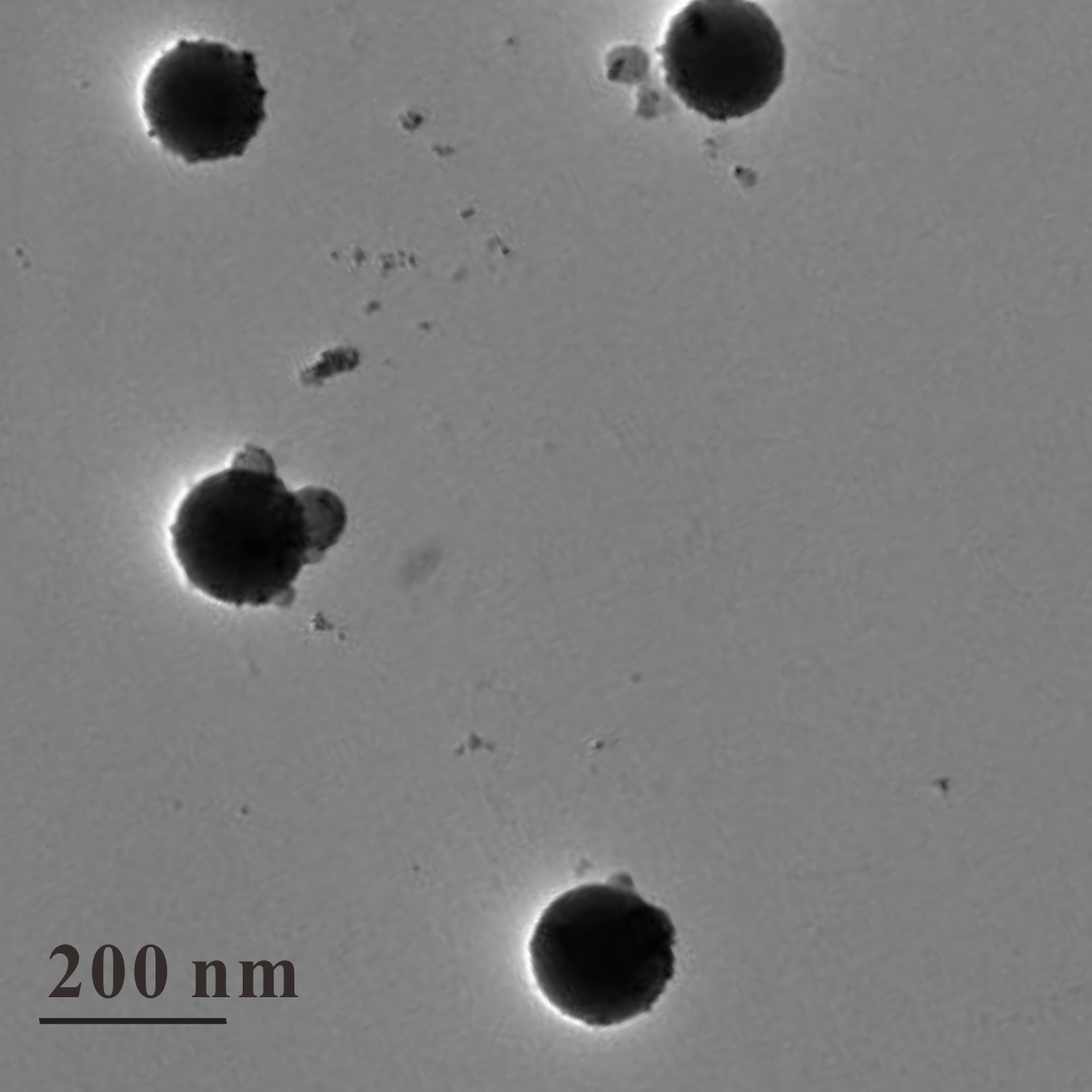

Supplement: Supplementary file 2 — Source Data [file 41467_2020_15016_MOESM2_ESM.zip › Source Data/Figure 1d.jpg]

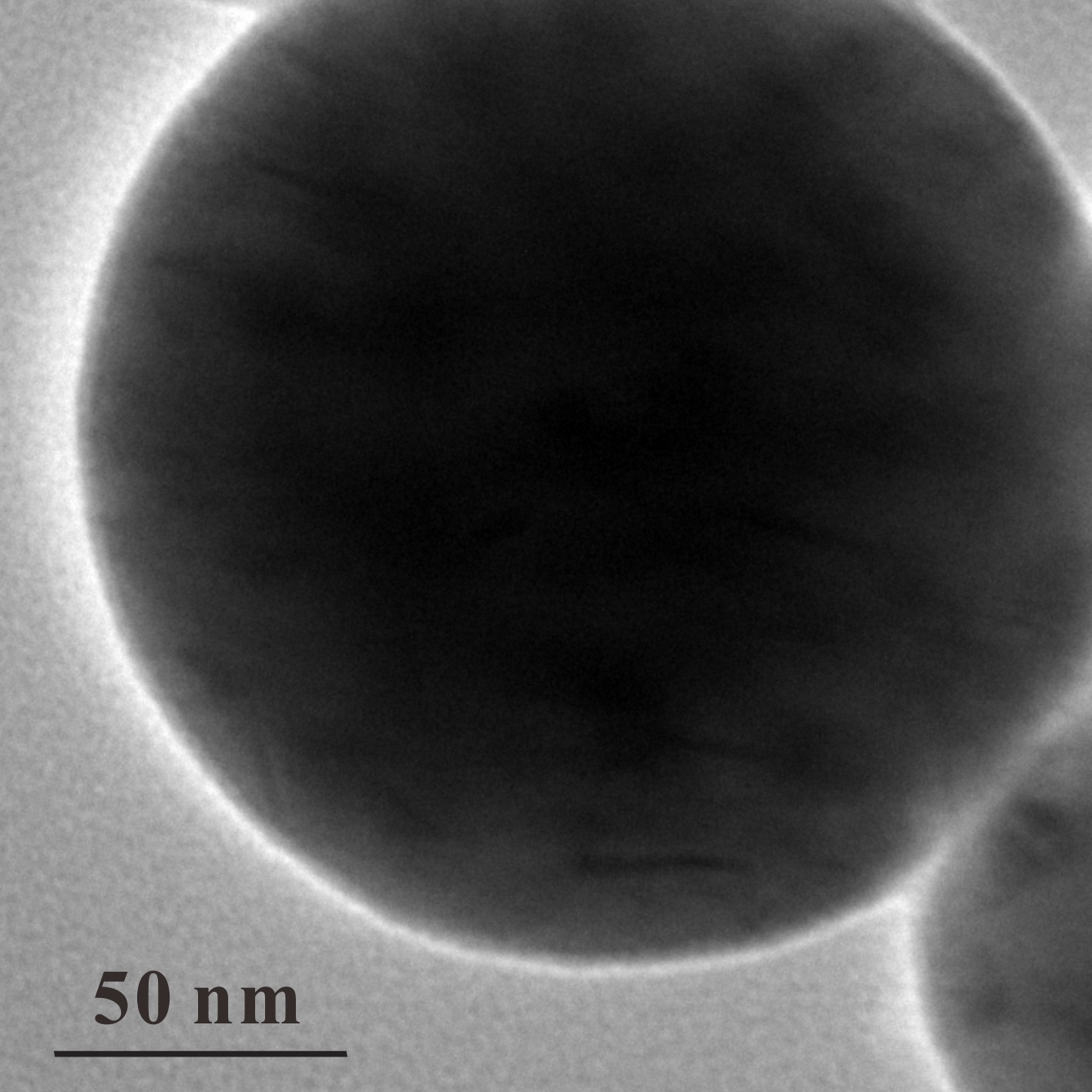

Supplement: Supplementary file 2 — Source Data [file 41467_2020_15016_MOESM2_ESM.zip › Source Data/Figure 1e.jpg]

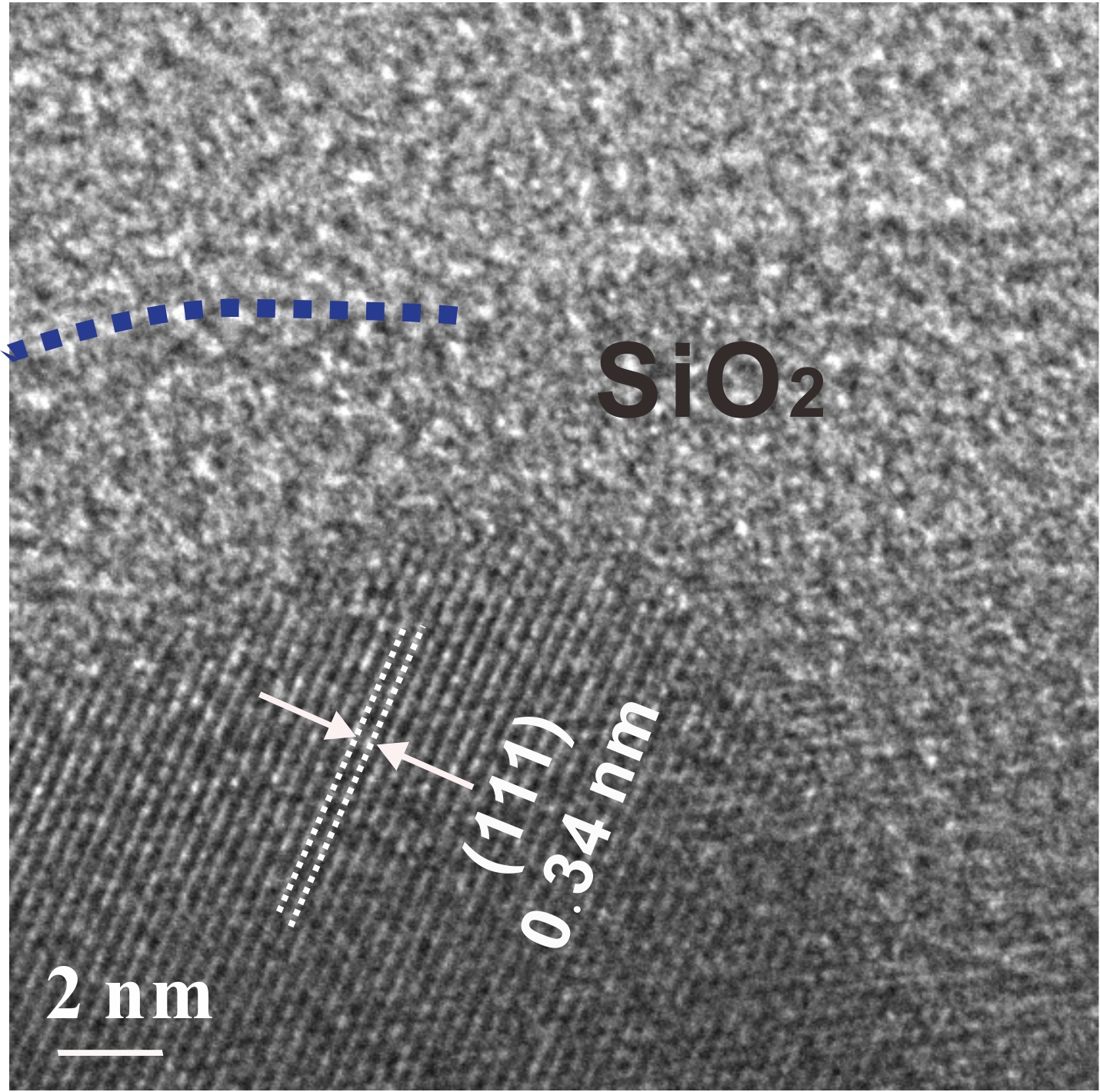

Supplement: Supplementary file 2 — Source Data [file 41467_2020_15016_MOESM2_ESM.zip › Source Data/Figure 1f.jpg]

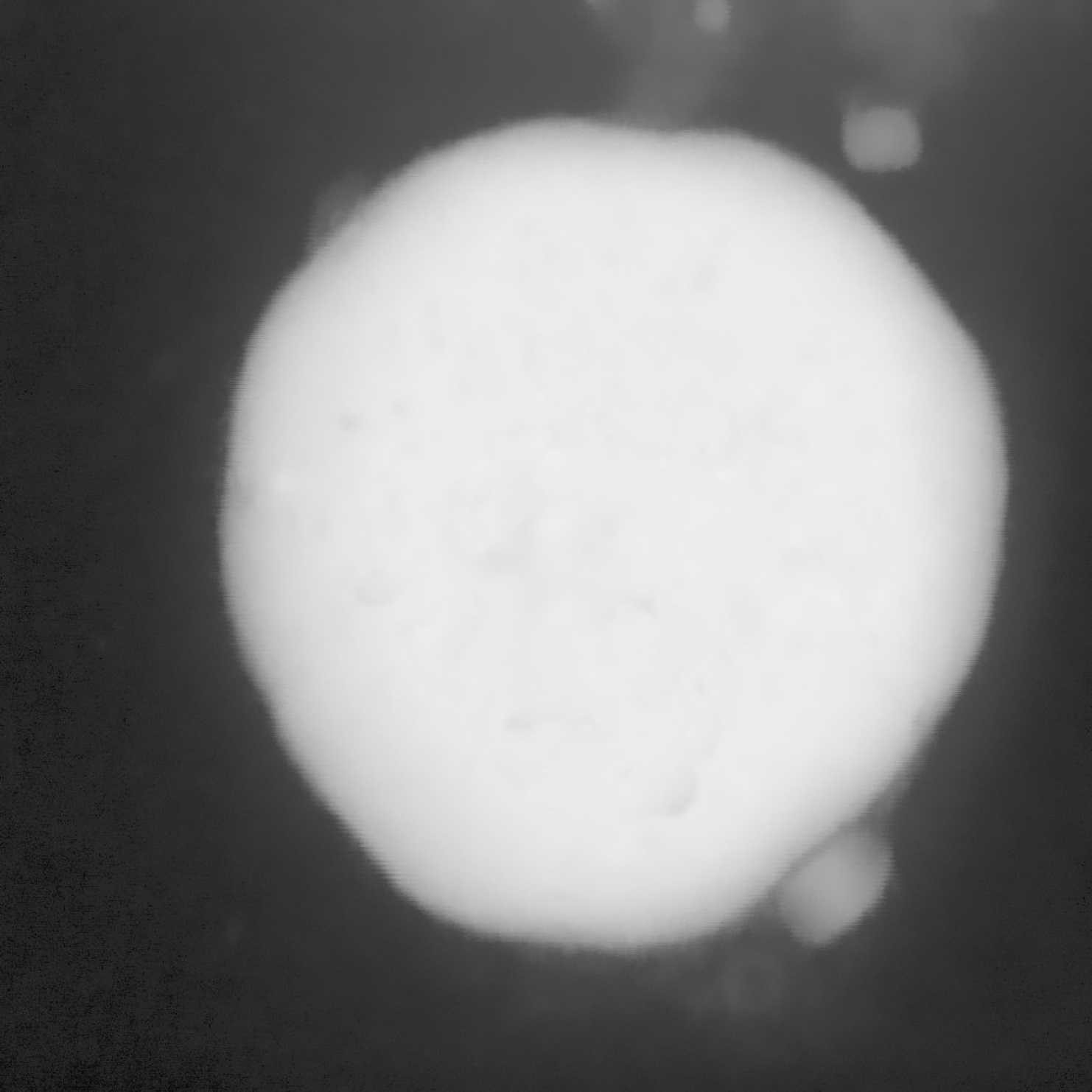

Supplement: Supplementary file 2 — Source Data [file 41467_2020_15016_MOESM2_ESM.zip › Source Data/Figure 1g-1.jpg]

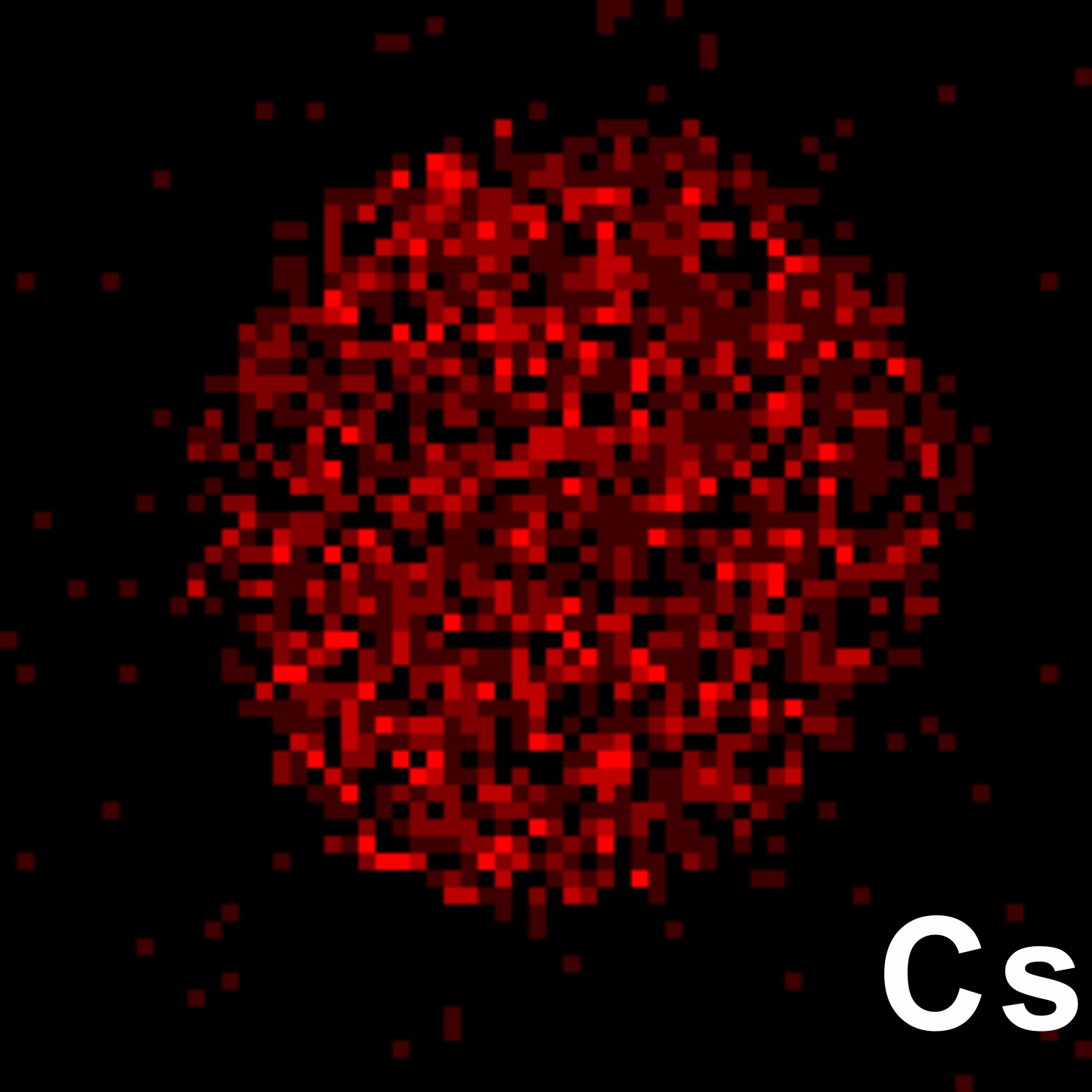

Supplement: Supplementary file 2 — Source Data [file 41467_2020_15016_MOESM2_ESM.zip › Source Data/Figure 1g-2.jpg]

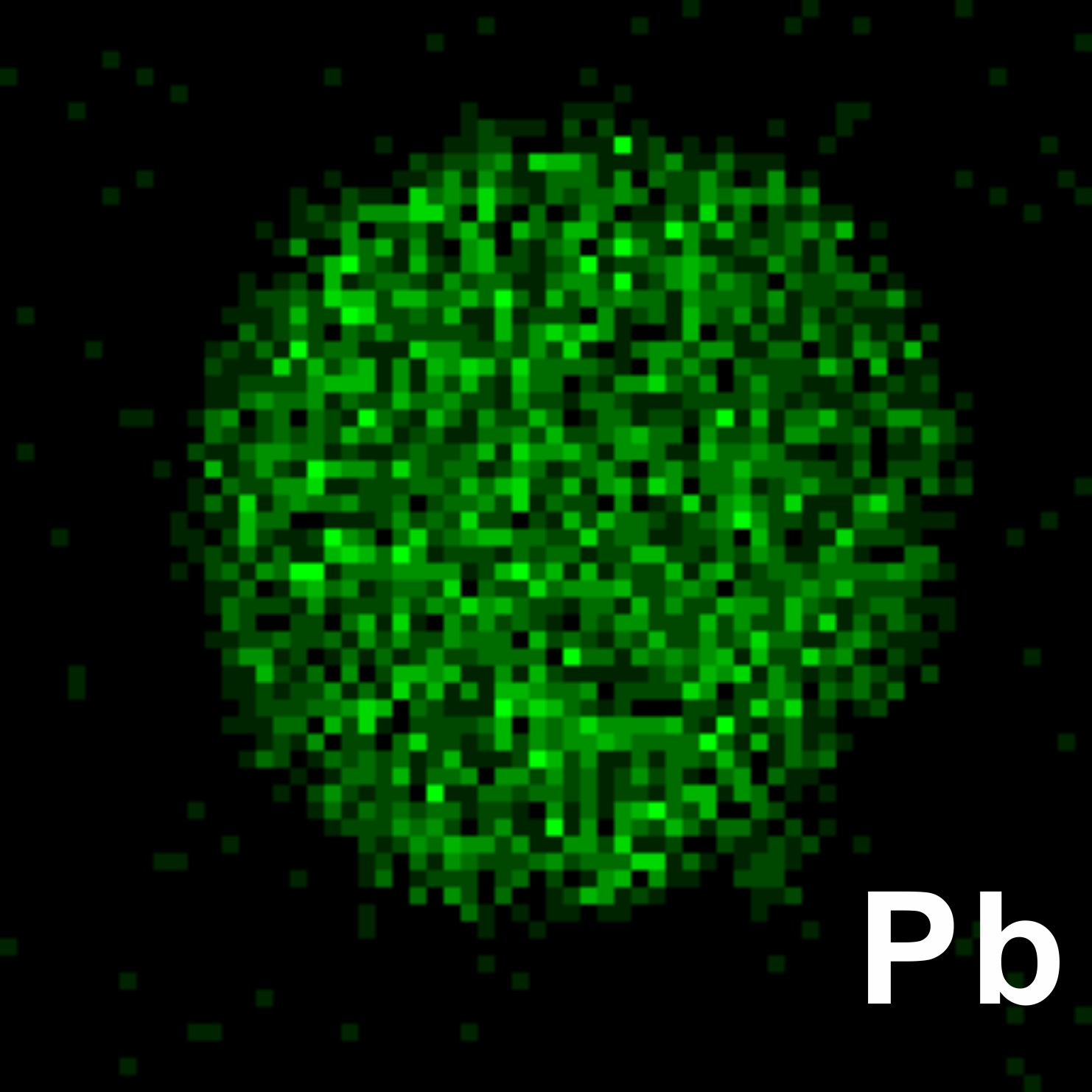

Supplement: Supplementary file 2 — Source Data [file 41467_2020_15016_MOESM2_ESM.zip › Source Data/Figure 1g-3.jpg]

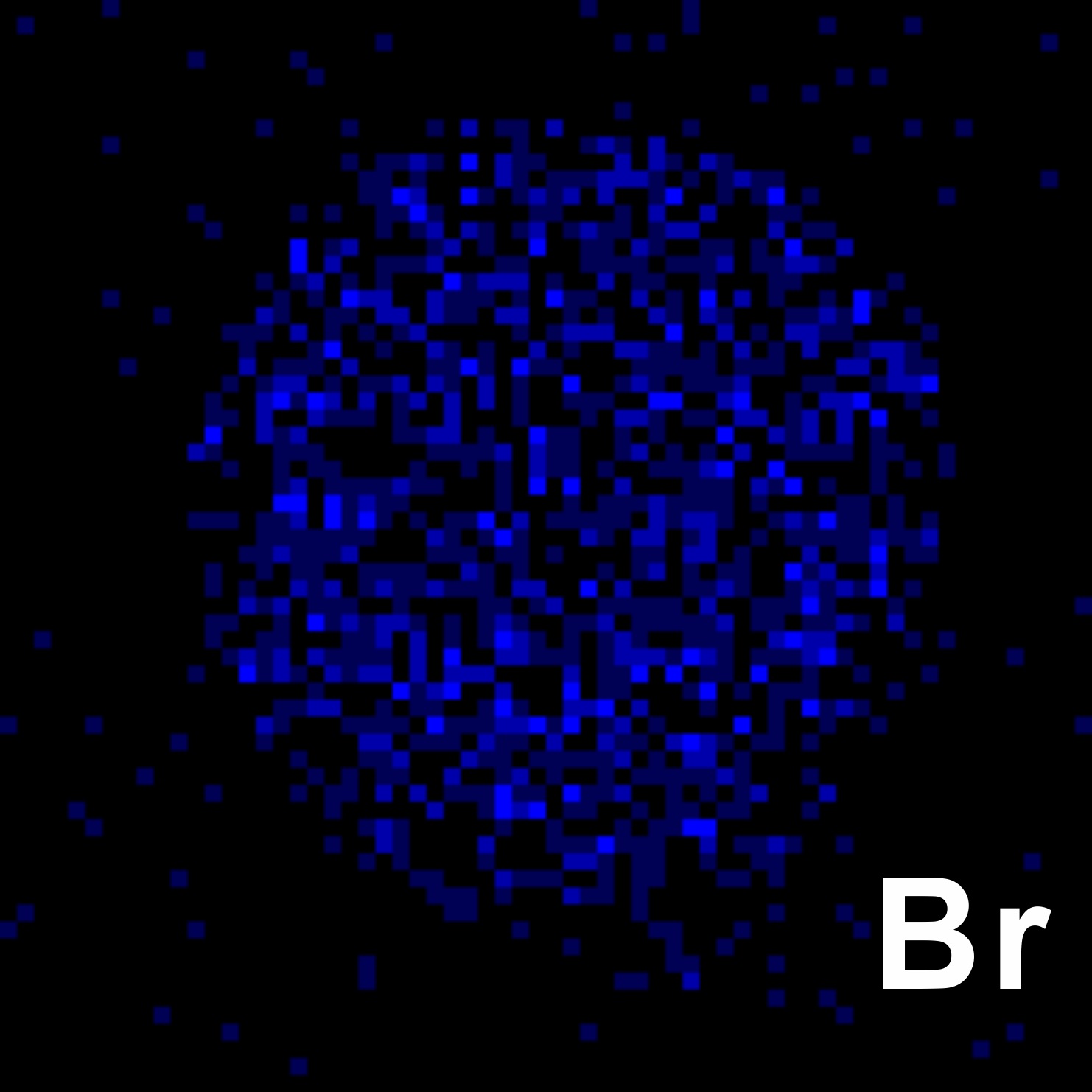

Supplement: Supplementary file 2 — Source Data [file 41467_2020_15016_MOESM2_ESM.zip › Source Data/Figure 1g-4.jpg]

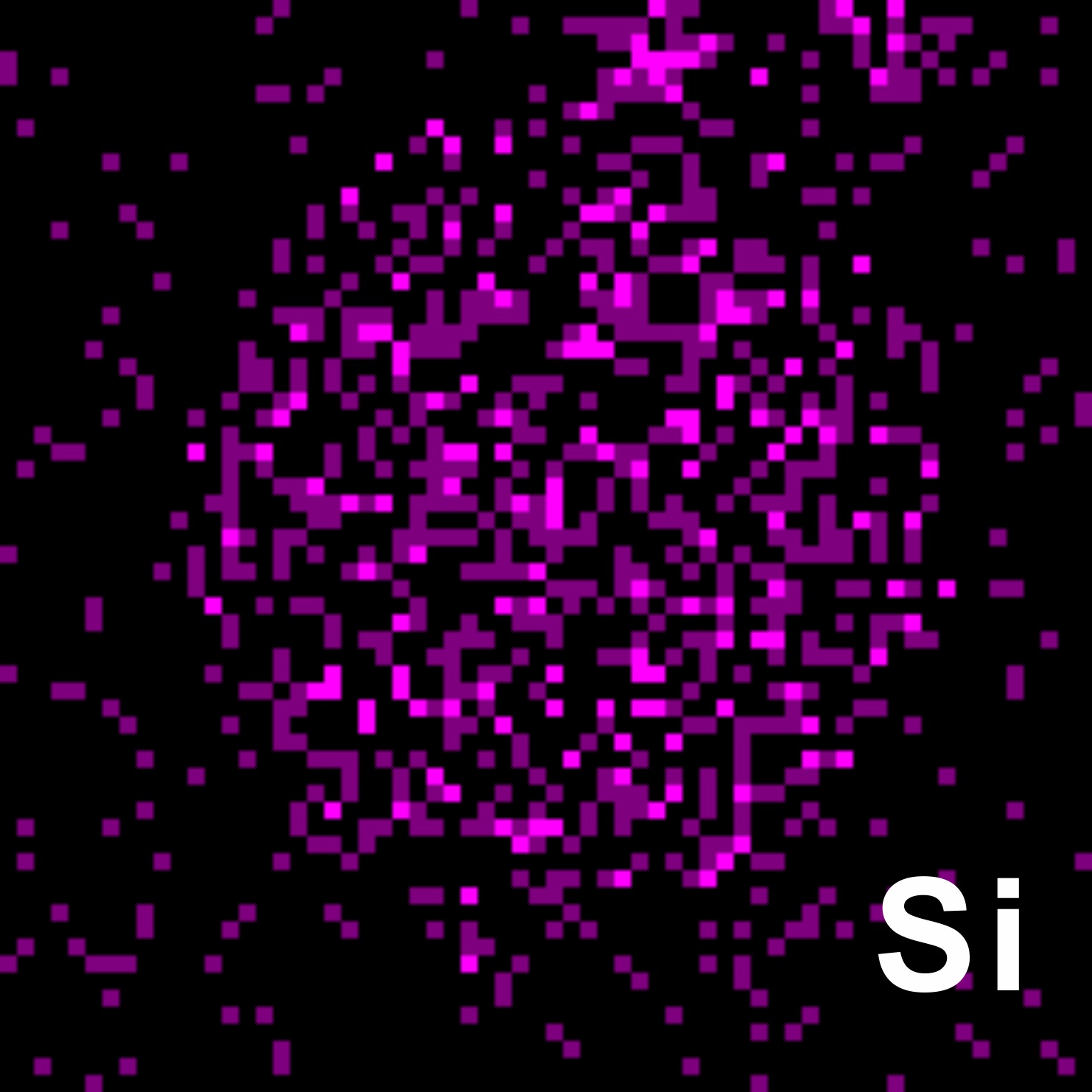

Supplement: Supplementary file 2 — Source Data [file 41467_2020_15016_MOESM2_ESM.zip › Source Data/Figure 1g-5.jpg]

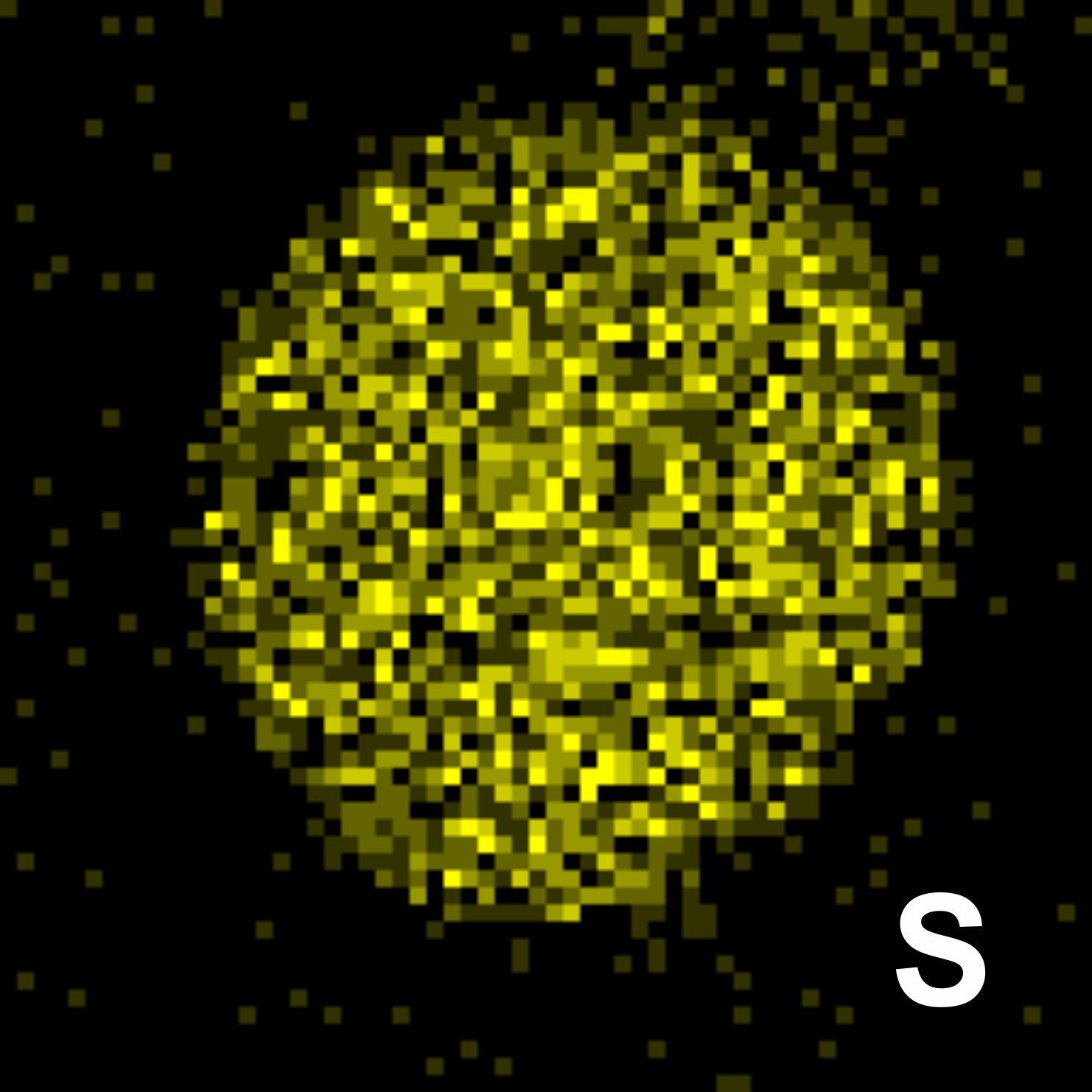

Supplement: Supplementary file 2 — Source Data [file 41467_2020_15016_MOESM2_ESM.zip › Source Data/Figure 1g-6.jpg]

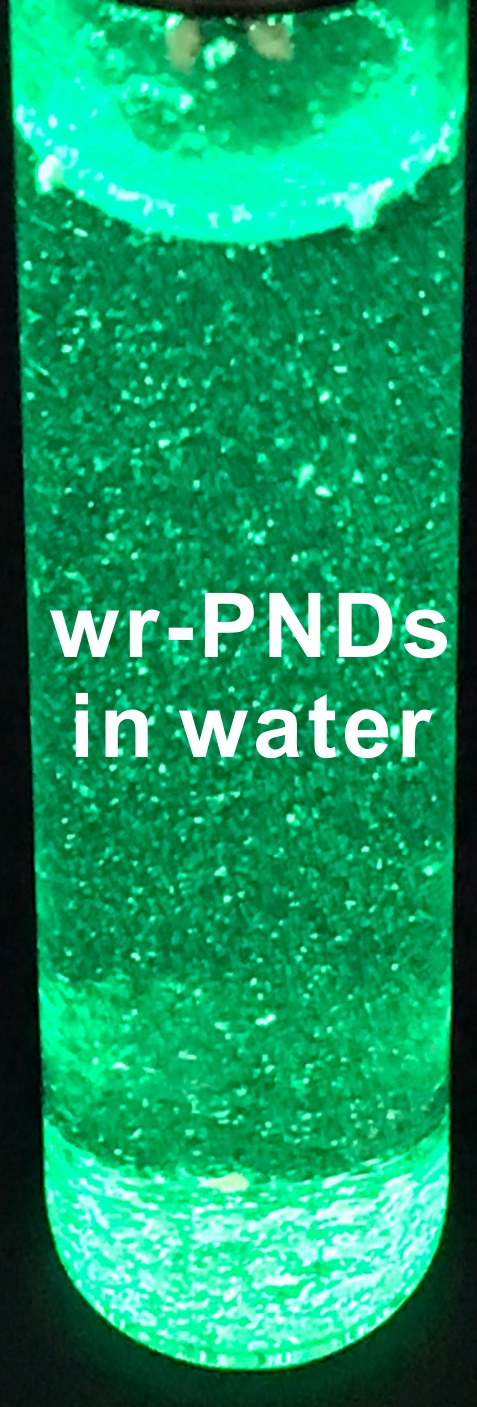

Supplement: Supplementary file 2 — Source Data [file 41467_2020_15016_MOESM2_ESM.zip › Source Data/Figure 1h.jpg]

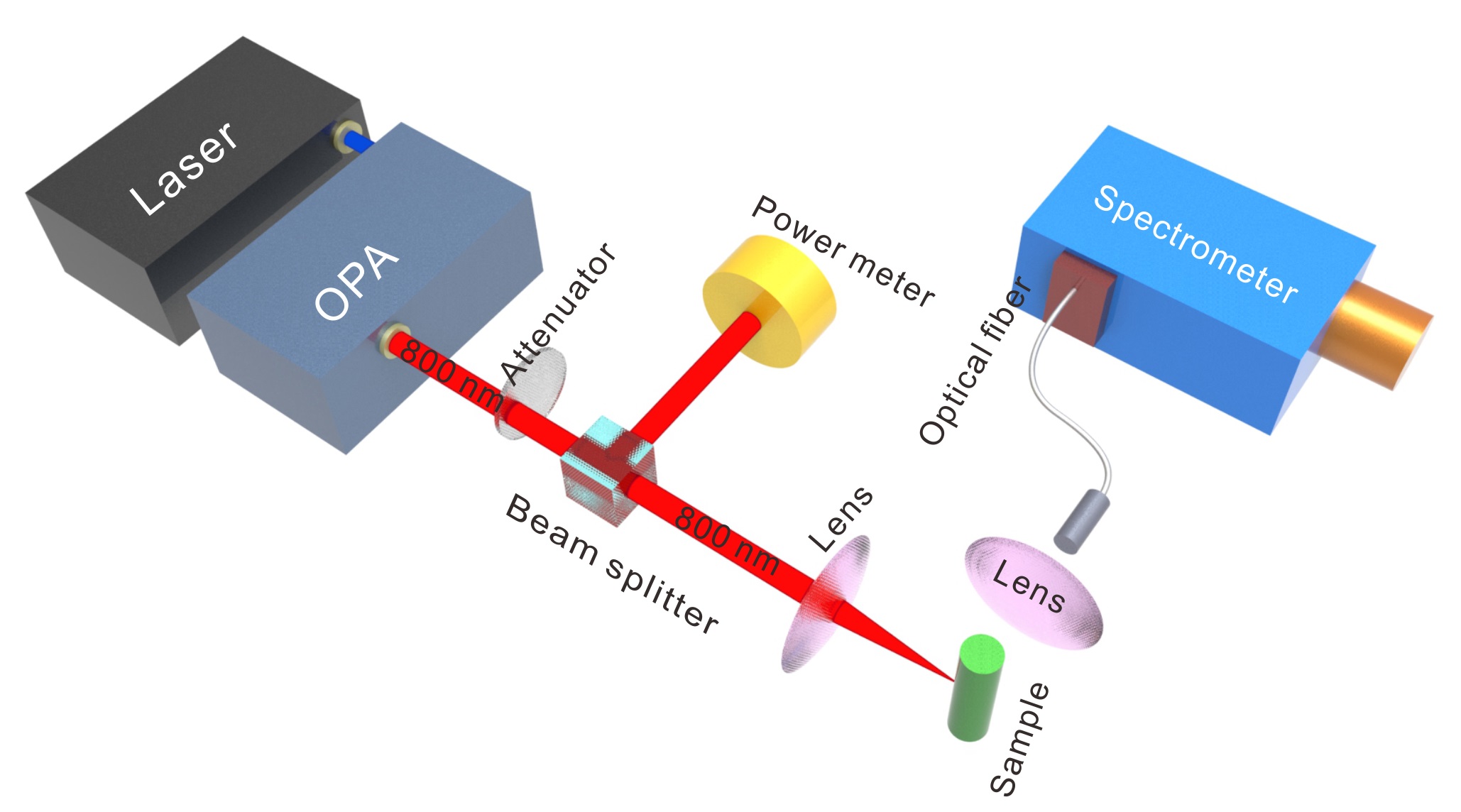

Supplement: Supplementary file 2 — Source Data [file 41467_2020_15016_MOESM2_ESM.zip › Source Data/Figure 4a.jpg]

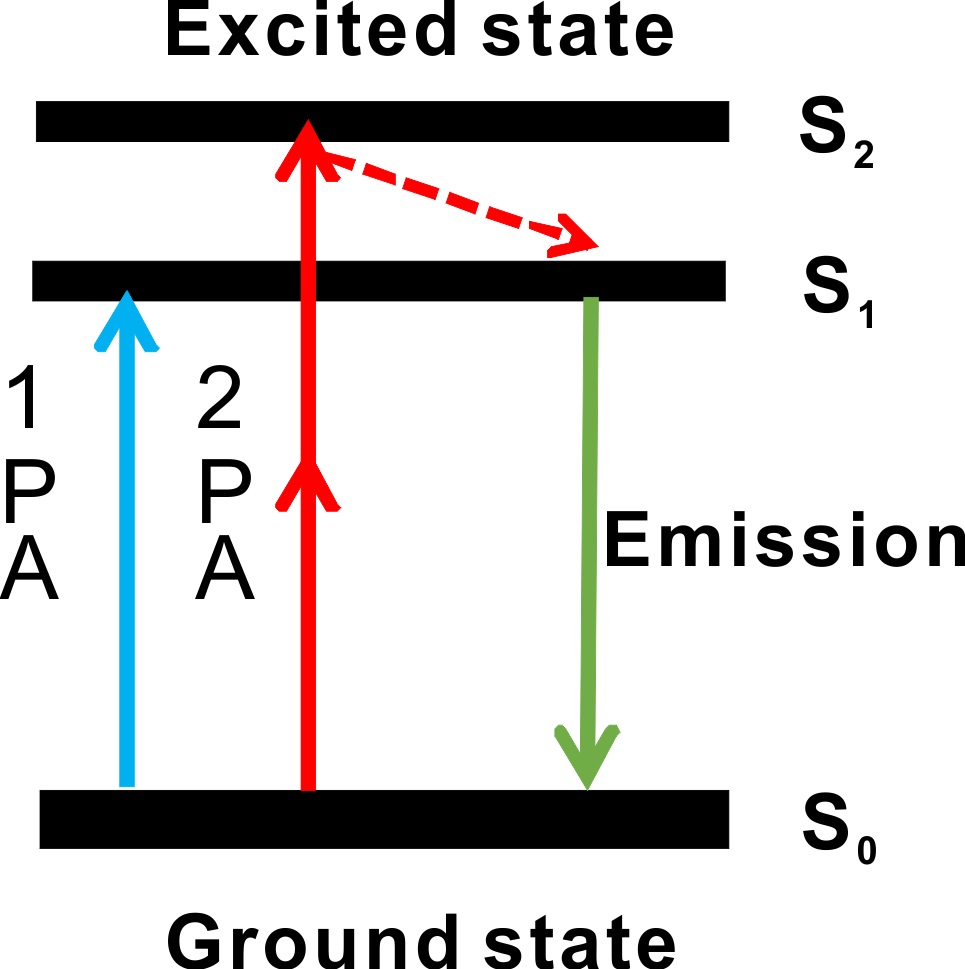

Supplement: Supplementary file 2 — Source Data [file 41467_2020_15016_MOESM2_ESM.zip › Source Data/Figure 4b.jpg]

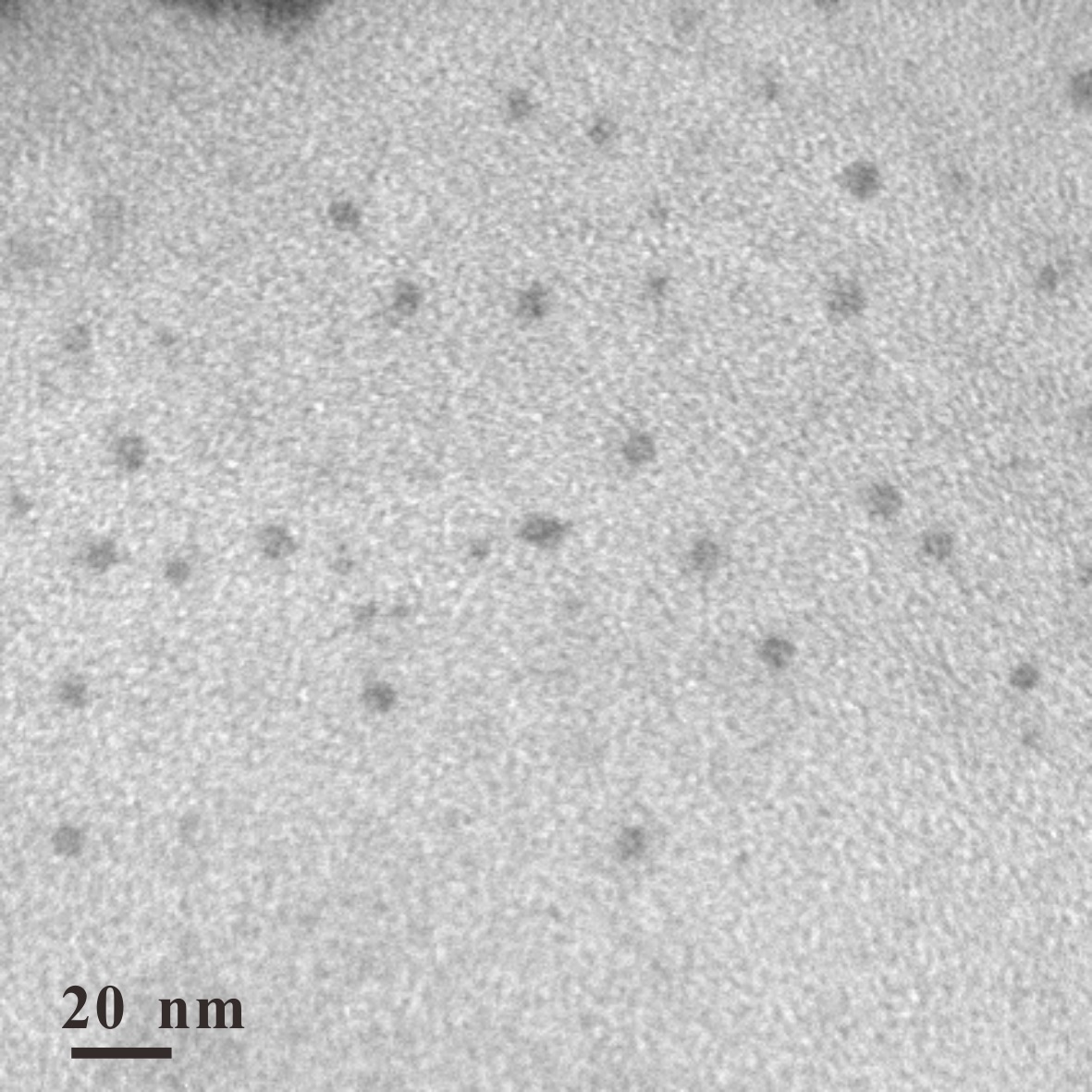

Supplement: Supplementary file 2 — Source Data [file 41467_2020_15016_MOESM2_ESM.zip › Source Data/Supplementary Figure 2a.jpg]

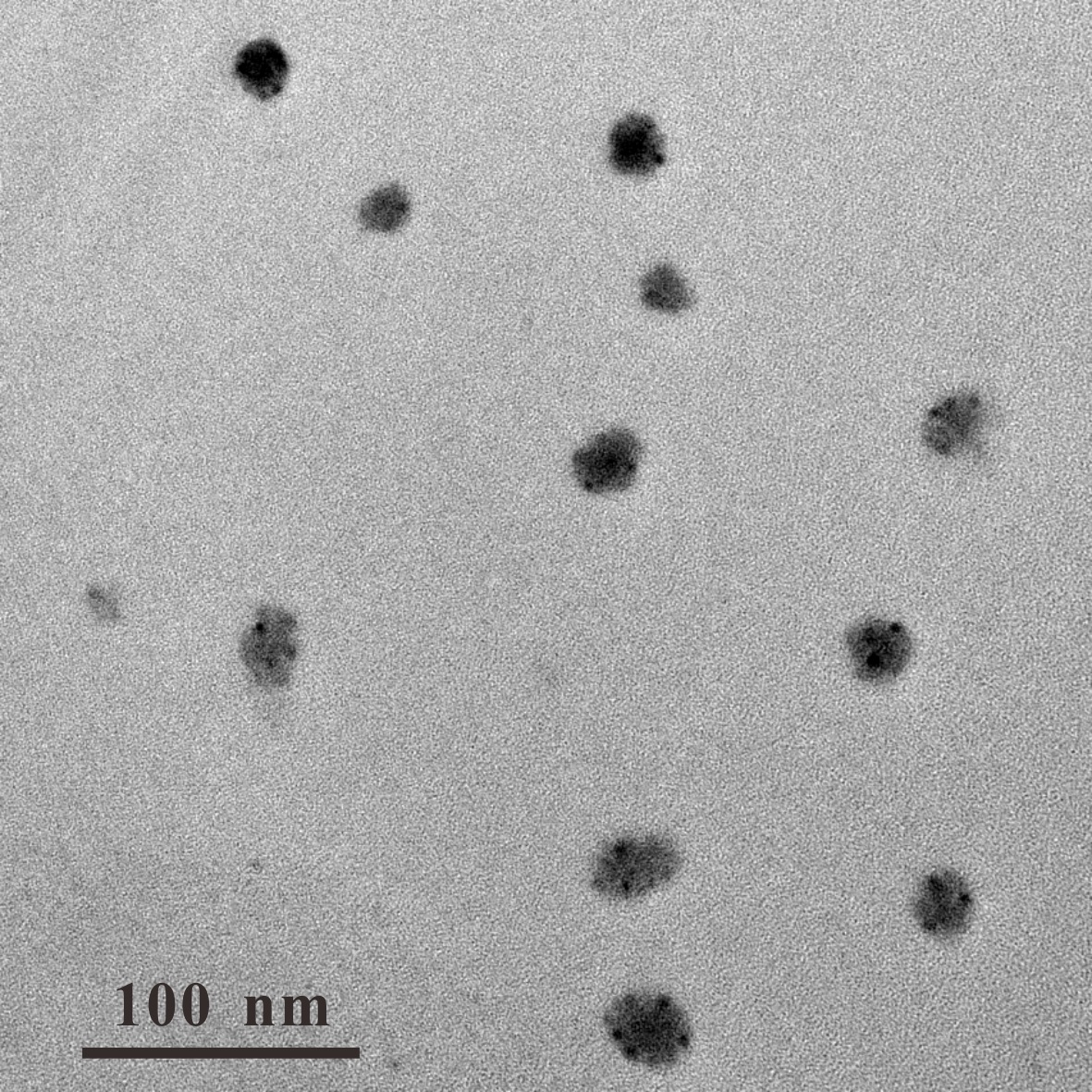

Supplement: Supplementary file 2 — Source Data [file 41467_2020_15016_MOESM2_ESM.zip › Source Data/Supplementary Figure 2b.jpg]

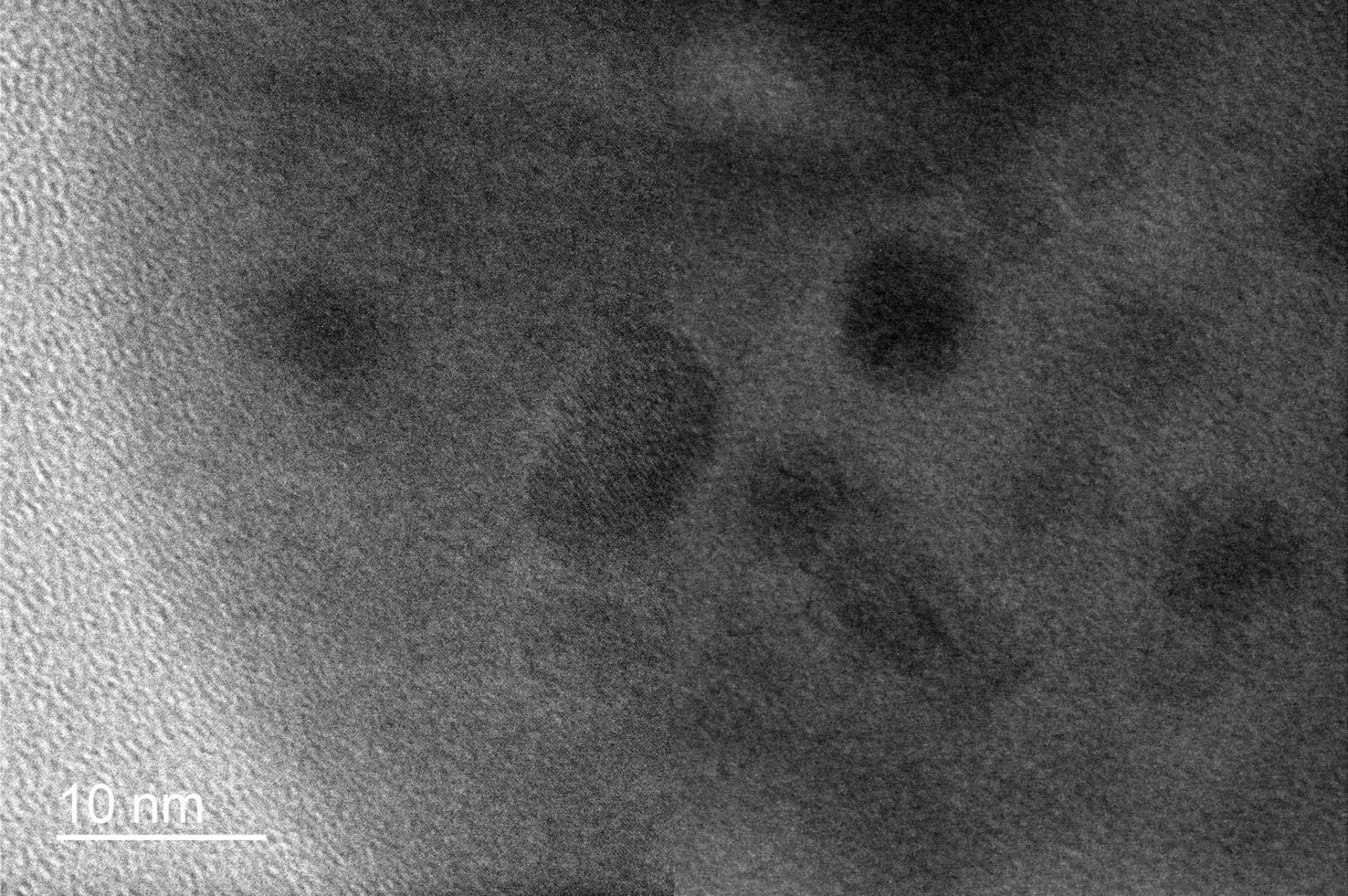

Supplement: Supplementary file 2 — Source Data [file 41467_2020_15016_MOESM2_ESM.zip › Source Data/Supplementary Figure 3.jpg]

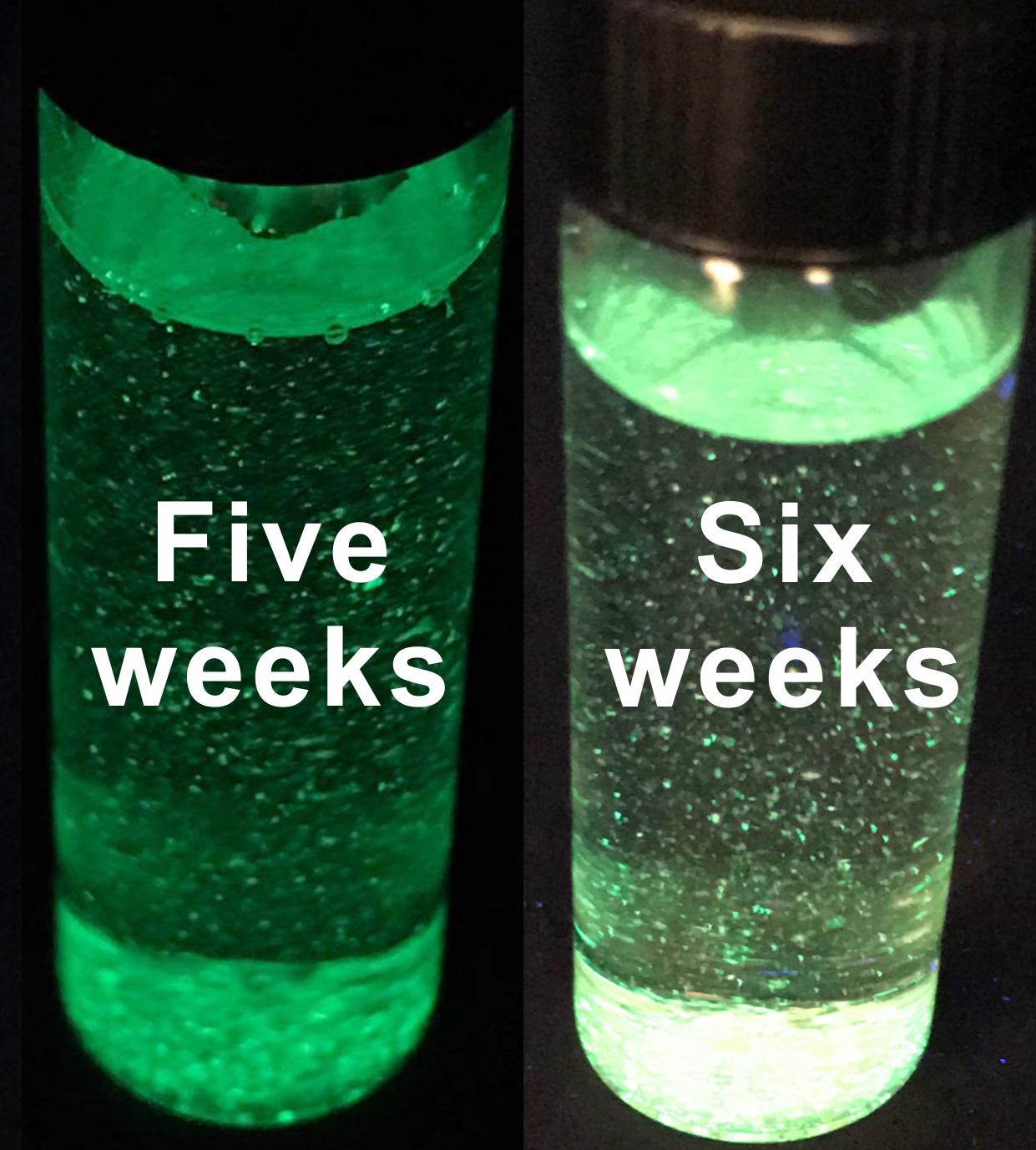

Supplement: Supplementary file 2 — Source Data [file 41467_2020_15016_MOESM2_ESM.zip › Source Data/Supplementary Figure 4.jpg]

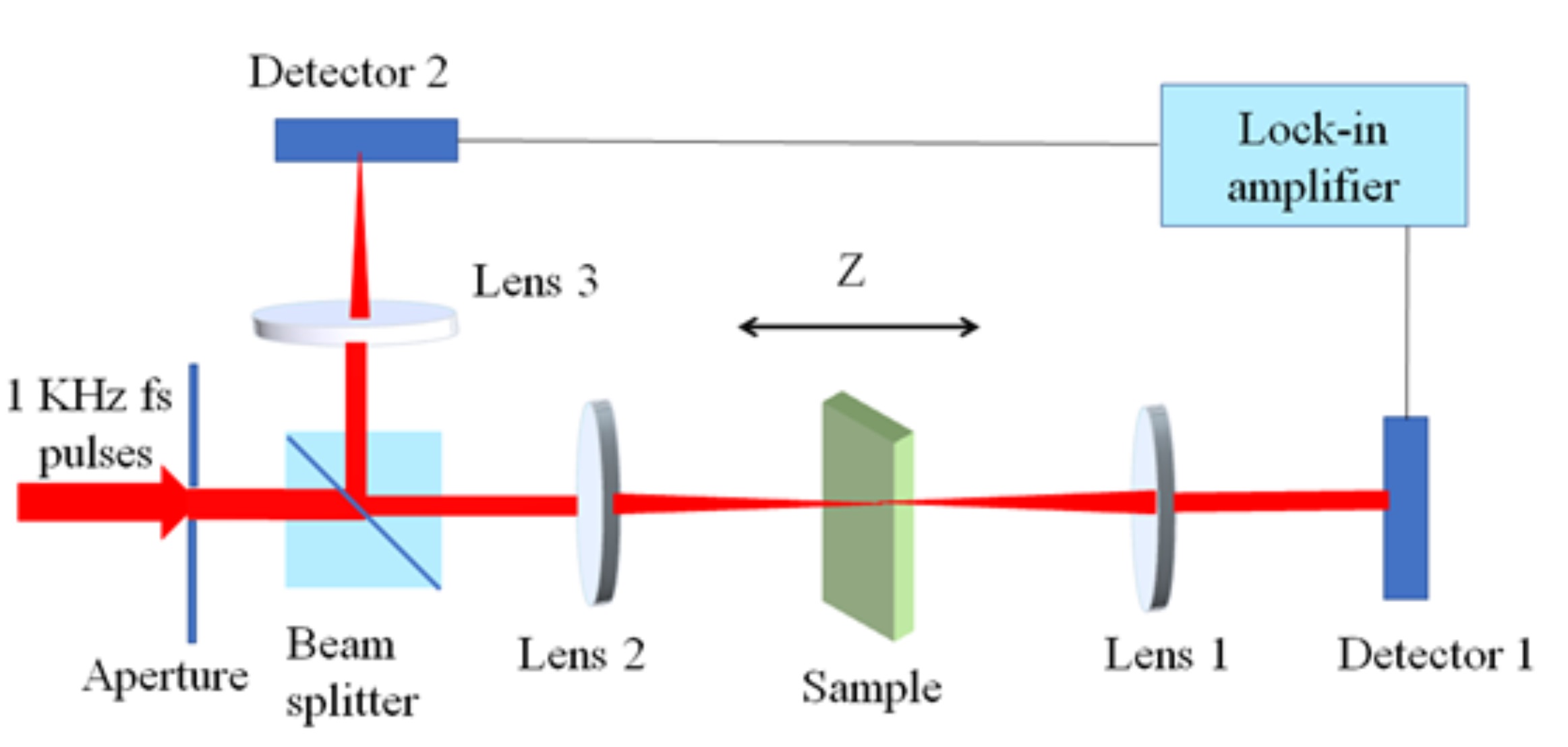

Supplement: Supplementary file 2 — Source Data [file 41467_2020_15016_MOESM2_ESM.zip › Source Data/Supplementary Figure 8.jpg]

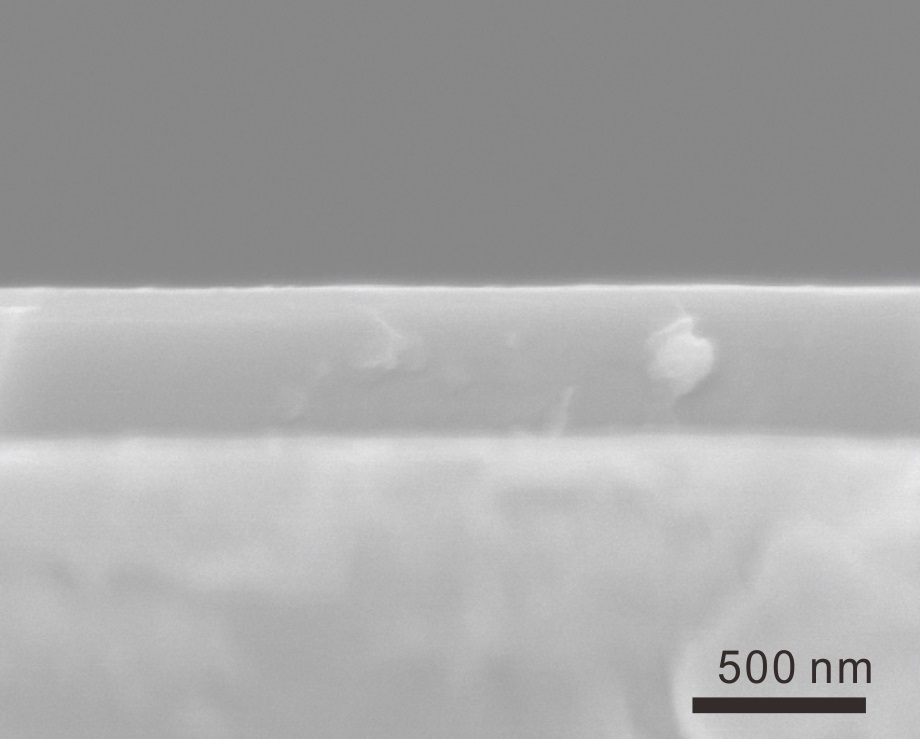

Supplement: Supplementary file 2 — Source Data [file 41467_2020_15016_MOESM2_ESM.zip › Source Data/Supplementary Figure 9a.jpg]

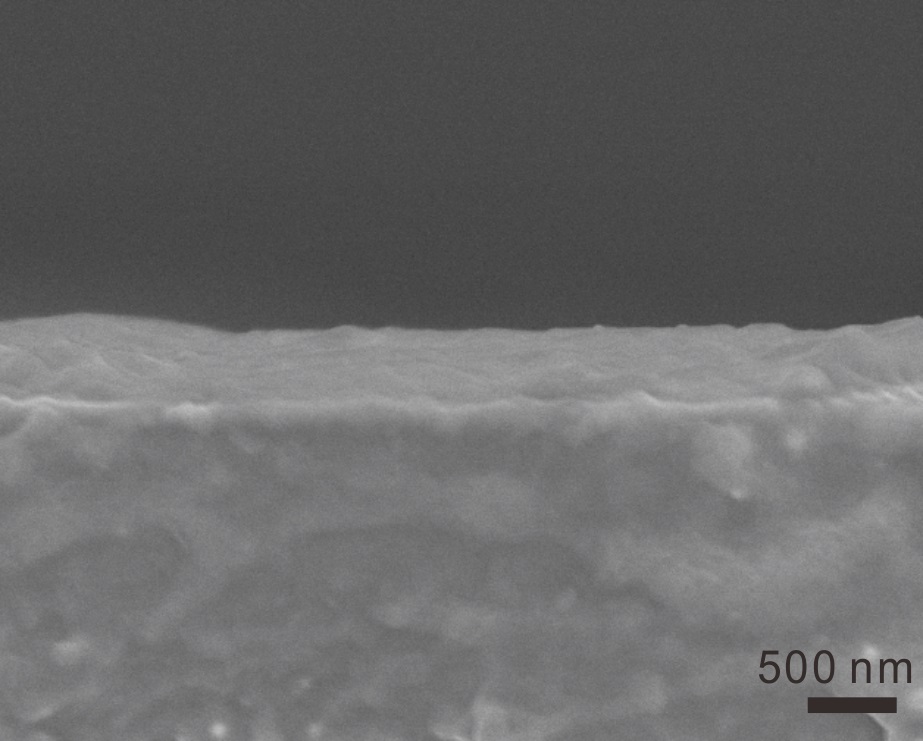

Supplement: Supplementary file 2 — Source Data [file 41467_2020_15016_MOESM2_ESM.zip › Source Data/Supplementary Figure 9b.jpg]
